# Predicting Postoperative Delirium in Older Patients Before Elective Surgery: Multicenter Retrospective Cohort Study

**DOI:** 10.2196/67958

**Published:** 2025-08-19

**Authors:** Shun-Chin Jim Wu, Nitin Sharma, Anne Bauch, Hao-Chun Yang, Jasmine L Hect, Christine Thomas, Sören Wagner, Bernd R Förstner, Christine A F von Arnim, Tobias Kaufmann, Gerhard W Eschweiler, Thomas Wolfers, PAWEL Study Group

**Affiliations:** 1 Department of Psychiatry and Psychotherapy, University of Tübingen, Tübingen, Germany; 2 German Center for Mental Health (DZPG), partner site Tübingen, Tübingen, Germany; 3 Department of Psychiatry, University of Cambridge, Cambridge, CB2 1TN, United Kingdom, 44 07423706114; 4 Department of Neurological Surgery, University of Pittsburgh School of Medicine, Pittsburgh, PA, United States; 5 Department of Geriatric Psychiatry and Psychotherapy, Klinikum Stuttgart, Stuttgart, Germany; 6 Department of Anesthesiology, Klinikum Stuttgart, Stuttgart, Germany; 7 Department of Anesthesiology and Intensive Care, School of Medicine and Health, Klinikum Rechts der Isar, Technical University of Munich, Munich, Germany; 8 Social and Preventive Medicine, Department of Sports and Health Sciences, University of Potsdam, Potsdam, Germany; 9 Department of Geriatrics, University Medical Center Göttingen, Göttingen, Germany; 10 German Center for Cardiovascular Research (DZHK), Göttingen, Germany; 11 Norwegian Centre for Mental Disorders Research, University of Oslo, Oslo, Norway; 12 Geriatric Center, Universitätsklinikum Tübingen, Tübingen, Germany

**Keywords:** postoperative delirium, predictive model, preoperative cognition, geriatrics, elective surgery

## Abstract

**Background:**

Elective surgeries for older adults are increasing. Machine learning could enhance risk assessment, influencing surgical planning and postoperative care. Preoperative cognitive assessment may facilitate early detection and management of postoperative delirium (POD).

**Objective:**

This study aims to assess machine learning models' predictive ability for POD, focusing on the added predictive value of the neuropsychological assessments before elective surgery.

**Methods:**

This retrospective cohort study analyzed data from the multicenter PAWEL (Patient safety, Efficiency and Life quality in elective surgery) and PAWEL-R (risk) studies, encompassing older patients (≥70 y) undergoing elective surgeries from July 2017 to April 2019. A total of 1624 patients (52.3% male, N=850; age: mean 77.9, SD 4.9 years) were included, with a POD diagnosis made before discharge. Sociodemographic, clinical, surgical, and neuropsychological features were collected pre- and intraoperatively by care providers. Machine learning models’ performance was evaluated using the area under the receiver operating characteristic curve (AUC), with permutation testing for significance, and Shapley Additive Explanations to identify effective neuropsychological assessments.

**Results:**

Predicting POD before surgery with a random forest model achieved an AUC of 0.760. Incorporating all pre- and intraoperative features into the model yielded a slightly higher AUC of 0.783, with no statistically significant difference observed (*P*=.24). While cognitive factors alone were not strong predictors (AUC=0.617), specific tests within neuropsychological assessments, such as the Montreal Cognitive Assessment and Trail Making Tests, showed high feature attribution and played a crucial role in further enhancing prediction before surgery.

**Conclusions:**

Preoperative risk prediction for POD can increase risk awareness in presurgical assessment and improve perioperative management in older patients at a high risk for delirium.

## Introduction

In an aging society, there is a rising demand for elective surgeries due to the changing health care needs of older people [1-3]. However, this increase in elective surgeries raises concerns about additional adverse outcomes, particularly given the unique challenges posed by aging, such as pre-existing health conditions, disease sequelae, and diminished physiological reserves [4,5]. Meeting the growing demand for elective surgeries among older adults requires a comprehensive strategy, including thorough preoperative assessments, personalized care plans, and continuous postoperative support [6-8].

Postoperative delirium (POD), characterized by acute and fluctuating inattention with alterations in thinking or consciousness after surgery, affects 12% to 51% of older patients, with incidence varying by surgical procedures and regions [9]. POD in older patients is linked to heightened rehospitalization rates, persistent postoperative cognitive dysfunction, increased incidence of dementia, and elevated mortality [10-12]. Some studies have shown significant associations between POD and factors collected before or during surgery (pre- or intraoperatively) [13,14]. These factors could aid in predicting the POD [15,16]. However, different pre- and intraoperative feature categories may vary in their importance for POD risk prediction. Sociodemographic factors, such as age and sex, are critical for assessing POD risk [17-24]. Clinical data, including blood samples and chronic disease medication, is also predictive [25-27]. The type and duration of surgery and anesthesia have been shown to be indispensable for POD prediction [18,28]. Although preoperative neuropsychological assessments, like the Mini-Mental State Examination (MMSE) and others [29-31], help identify at-risk patients for early risk mitigation [32-34], these evaluations are not yet incorporated into clinical routine despite experts’ recommendations [35]. The early identification of POD predictors enables clinicians to proactively assess risks to mitigate the occurrence of POD [36] and might affect patients’ decisions before nonemergent surgery. Therefore, identifying preoperative predictors for POD is crucial in facilitating personalized surgical risk assessment before surgery [37,38]. Moreover, examining POD risks remains challenging due to its multifactorial origins [18,22,39]. The precise categories of preoperative and intraoperative features with superior predictive capabilities for POD remain unknown and unvalidated [40].

In this study, we (1) predict POD by using machine learning approaches using diverse pre- and intraoperative features from a large multicenter cohort of older patients and (2) conduct a comparative analysis of independent models using various predictor categories, including sociodemographic, clinical, surgical, and neuropsychological features. By featuring one of the largest cohorts to date, this multicenter study provides an extensive assessment of POD predictors in both cardiac and noncardiac surgeries through machine learning techniques. This approach can enhance presurgical risk assessment and perioperative management in older patients.

## Methods

### Participants

Building on previous research [13,41,42], this study comprehensively investigates the predictive capabilities of various feature categories of the entire cohort from the PAWEL (Patientensicherheit, Wirtschaftlichkeit und Lebensqualität bei elektiven Operationen, English: Patient safety, Efficiency and Life quality in elective surgery) study, originally recruited between November 21, 2017, and April 12, 2019. In addition, it also includes additional participants from the add-on PAWEL-R study (R for risk estimation), which extended the recruitment period from July 11, 2017, to January 15, 2019. By incorporating both datasets, our study expanded the cohort size, strengthening the predictive model’s reliability. Machine learning models were used to predict POD, departing from original statistical non–cross-validated methods in the previous reports [13]. Patients were recruited from 5 major medical institutions in Germany (3 university hospitals: Tübingen, Freiburg, and Ulm, and 2 tertiary medical centers: Stuttgart and Karlsruhe). The study adheres to CONSORT-EHEALTH (Consolidated Standards of Reporting Trials of Electronic and Mobile Health Applications and Online Telehealth) [43] and STROBE (Strengthening the Reporting of Observational studies in Epidemiology) [44] guidelines (the CONSORT-EHEALTH checklist is provided in Checklist 1). This study was approved by ethical votes under the PAWEL-R study, and ethical approval (Ethical Committee 233/2017BO1) was provided by the Ethical Committee of Tübingen University Hospitals, Tübingen, Germany on June 6, 2017.

Participants included patients aged 70 years and older undergoing elective surgery (joint, spine, vessels, heart, lung, abdomen, urogenital system, and other organs) with an expected surgical duration exceeding 60 minutes. Exclusion criteria covered patients unable to communicate effectively in German, those undergoing emergency surgery, severe dementia with MMSE <15 or Montreal Cognitive Assessment (MoCA) <8, or an estimated survival time of less than 15 months. A stepped-wedge cluster randomized design was used for equitable intervention group allocation [41,42]. The study, initially including 1631 patients, conducted thorough postoperative assessments within 1 week after surgery but before discharge. To ensure accuracy in predicting POD diagnosis, patients without sufficient information for POD diagnosis before discharge (N=7) were excluded, resulting in 1624 patients included in prediction models (Figure S1). The study reported 23.1% of patients diagnosed with POD, with detailed between-group comparisons available in Tables S1 and S2.

### Measures

The PAWEL and PAWEL-R studies extensively evaluated elective surgery patients to identify risk factors and outcomes related to POD. Diagnosis involved the Confusion Assessment Method (I-CAM) algorithm and chart review within the first postoperative week or until discharge. Features were categorized into 4 feature groups: sociodemographic, neuropsychological, clinical, and surgical information.

Sociodemographic data, including age, sex, education, alcohol and smoking habits, living arrangements, and hospital location, were collected preoperatively. Neuropsychological assessments such as MoCA, Trail Making Test (TMT) parts A and B, digit span backward, Subjective Memory Impairment (SMI), and Patient Health Questionnaire-4 (PHQ-4) were conducted during admission. Clinical profiles included blood samples, past medical histories (including pre-existing mild or moderate dementia and previous delirium history), preoperative and intraoperative medication dose (including benzodiazepine, neuroleptics, opiates, and propofol), polypharmacy, multimorbidity, the mininutritional assessment, American Society of Anesthesiologists (ASA) physical status classification system, Charlson comorbidity index, Barthel index, clinical frailty scale, and sensory impairments (auditory, visual, and others). Surgical information covered types and duration of surgery and anesthesia, cardiopulmonary bypass, and intraoperative physiological changes. Preoperative and intraoperative features were defined to assess predictive performance achieved at 2 distinct time points: before and during surgery. Additional details can also be found in Table S1.

To identify effective predictors for POD diagnosis, the specific and combined predictive capacity of sociodemographic, clinical, and surgical features before (preoperative) and during surgery (intraoperative) were analyzed. Models were developed and tested with both features and preoperative features alone. In addition, models were compared with and without neuropsychological assessments to examine their contribution to predictive performance.

### Preprocessing, Imputation, and Features

Two features with excessive missingness were excluded, namely, albumin level (979/1624, 60.28%) and depth of anesthesia (1224/1624, 75.37%) were excluded [16]. Information about missing values is available in Table S3. Data imputation involved using multiple imputation (IterativeImputer) for continuous variables and random sampling from the original probability distribution for discrete and binary variables within cross-validation folds for all models, except for gradient boosting, which does not require imputation [45]. Continuous and discrete variables were scaled within cross-validation folds for all models. Potential outliers or data entry errors in clinical assessments were identified when a data point exceeded 5 SD from the mean and were subsequently imputed using the above method.

Blood samples, including hemoglobin, sodium levels, and C-reactive protein (CRP), were interpreted and discretized following clinical guidelines from Harrison’s Principles of Internal Medicine, 20th version, in line with standard practice [46]. Specifically, hemoglobin levels of less than 12 g/dL were considered indicative of anemia, while values of 12 g/dL or higher were considered normal. Serum sodium levels were categorized as hyponatremia if below 135 mmol/L and hypernatremia if above 145 mmol/L, with values within this range considered normal. Similarly, CRP levels greater than 3 mg/L indicated elevated levels in adults aged 65 years and older [47], while values within the normal range were not considered clinically significant. Redundant features with a perfect correlation were excluded; therefore, use of heart-lung machine was excluded for its perfect correlation with cardiopulmonary bypass. Nonbinary categorical features, including location, SMI, types of anesthesia and surgery, and transformed blood samples were one-hot encoded because they had no natural ordinal relationship among their categories, and assigning numerical labels to them could introduce bias or incorrect assumptions in the model.

Fifty-nine features were used, divided into preoperative (48 features) and intraoperative (11 features) categories. Preoperative features encompassed sociodemographic (7 features), clinical (20 features), surgical categories (6 features), and neuropsychological assessments (15 features). Intraoperative features included clinical (4 features) and surgical (7 features) categories. Different combinations of features were compared, including preoperative only, preoperative and intraoperative, and each category of preoperative features. The study evaluated model performance, feature category effectiveness, and the additional benefits of preoperative neuropsychological assessments.

The study aimed to develop a prediction model in a naturalistic setting. Information regarding interventions was included only as a sensitivity analysis to demonstrate its potential impact on the prediction model. The potential imbalance of the dataset for all models was tested by oversampling with the Synthetic Minority Oversampling Technique [48]. Given that the overall sex distribution in the cohort was fairly balanced (774/1624, 47.66% female), we did not apply additional rebalancing for sex to avoid potential bias. In addition, no significant age differences were observed between patients with and without POD. Various sensitivity analyses described were performed.

### Machine Learning Models, Performance Evaluation, and Feature Importance

**Figure 1.**
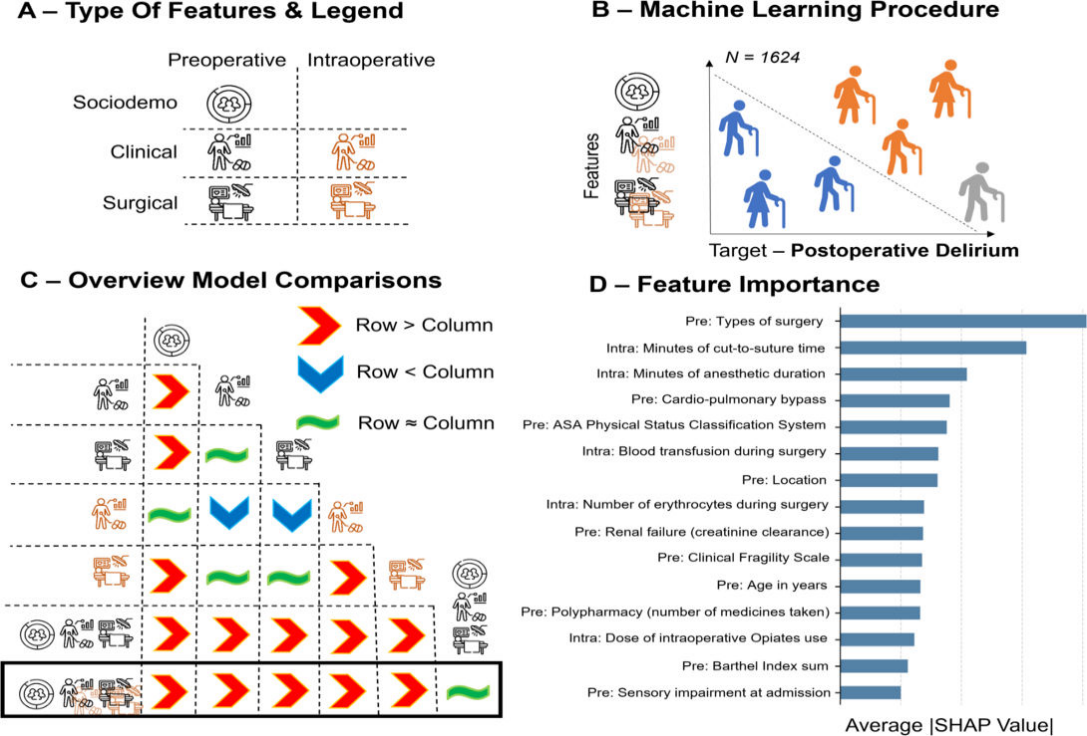
Individual feature categories perform worse than combined feature categories pre-, intraoperatively, and postoperative delirium (POD) can effectively be predicted prior to surgery (see back box). (**A**) The symbols are consistently used in this study and represent the different types of features. (**B**) A random forest classifier with 5-fold cross-validation was used to forecast POD onset in 1624 older individuals. Among them, 23.1% experienced POD. Orange indicates patients with POD, blue represents those without POD, and gray signifies patients predicted to have POD by the algorithm. (**C**) A comparative analysis of different feature combinations based on their respective area under the receiver operating characteristic curve (AUC) values, calculated over 1000 permutations was conducted. Combined feature sets consistently outperformed individual feature types. (**D**) Shapley Additive Explanations (SHAP) was used to assess feature importance. The feature importance was based on the average absolute value of the aggregated SHAP values across folds. Among preoperative and intraoperative features, surgical data is critical for predicting POD.

Machine learning models were used to predict POD, including logistic regression, support vector machines, random forest, and gradient boosting without hyperparameter tuning using the scikit-learn library version 1.2.2 [49] and the Xgboost library version (1.7.3) [50]. Independent variables were feature variables, while the dependent variable was POD diagnosis, as illustrated in Figure 1B. Feature selection was performed using the SelectFromModel function from scikit-learn, leveraging model-based feature selection, to retain the most predictive variables. Given the sample size relative to the complexity of the models, we did not anticipate substantial improvements from hyperparameter tuning. However, we conducted additional sensitivity analyses to assess its impact on model performance via nested cross-validation (Tables S12-S15 in Multimedia Appendix 1). To ensure model stability and interpretability, we used default parameters from the software for the primary analysis (Table S11 in Multimedia Appendix 1). 5-fold cross-validation, with balanced labels across folds, measured model performance at testing using the area under the receiver operating characteristic curve (AUC) as the primary metric. In order to robustly evaluate model performance against chance and between models, permutation testing was used to assess whether the AUC of each model was significantly greater than expected by chance. Specifically, POD diagnostic labels were randomly shuffled, and AUC values were recalculated 1000 times to generate a null distribution. The *P* value was derived by comparing the observed AUC to this null distribution (Table S4 in Multimedia Appendix 1). Furthermore, to compare AUC differences between models, permutation testing was also used to generate a null distribution of AUC differences by randomly permuting the labels 1000 times while preserving data dependencies. The *P* value for model comparison was computed based on the observed difference relative to this null distribution [51,52]. Lastly, to obtain a robust measure of variability in model performance, 95% CI for AUC using bootstrapping with 1000 resamples was estimated. Additional metrics included precision, recall, sensitivity, specificity, balanced accuracy, and area under the precision-recall curve presented in Table S5 in Multimedia Appendix 1.

The Shapley Additive Explanations (SHAP) values were used to assess feature importance. Positive SHAP values increased the probability of POD, while negative values decreased it. SHAP values were computed across all cross-validation folds and aggregated over 5-fold cross-validation with 5 shuffles to ensure stability and robustness in feature importance rankings. To facilitate explanation of the main models, we presented the top 15 features contributing to model performance and complete feature attributions in major feature sets: preoperative features with and without neuropsychological assessments, and combined preoperative and intraoperative features with and without neuropsychological assessments (Figures1D  2D, Figure S2 in Multimedia Appendix 1, and Tables S7-S10 in Multimedia Appendix 1). The SHAP library version 0.41.0 in Python was used [53]. To evaluate the calibration of our models, calibration plots were generated (applying Platt scaling if needed), and the number of patients with high-confidence positive predictions (>0.9) and high-confidence negative predictions (<0.1) was calculated for each model. This analysis was conducted across different feature sets to assess whether the addition of features affected the model’s probability estimates.

All preprocessing steps and analyses are available on GitHub upon publication from Sharma forked to our laboratory’s page, and data can be requested by addressing the PAWEL consortium as indicated in the data availability section.

**Figure 2.**
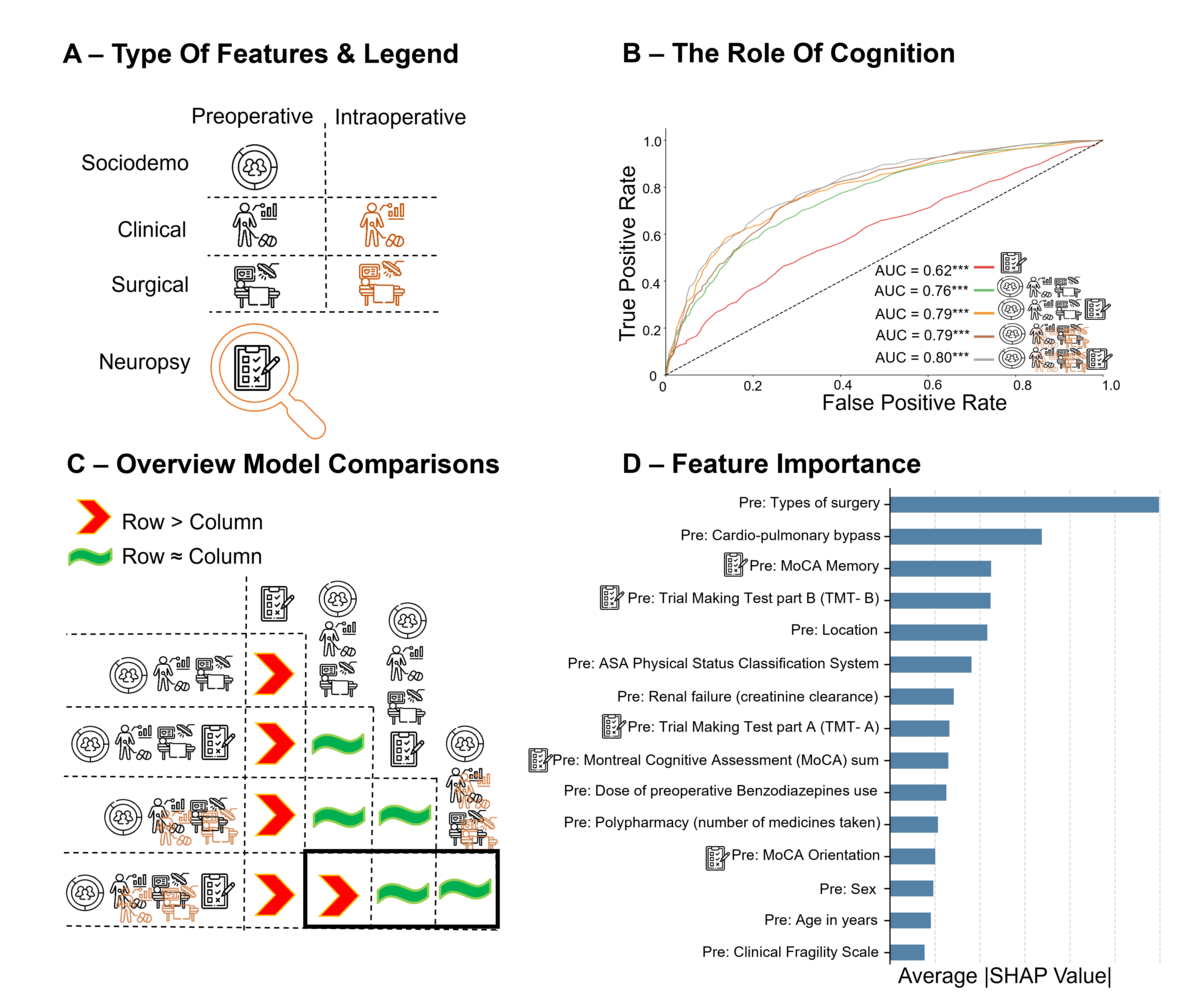
Cognition, in isolation, demonstrates limited predictive power. However, when combined with preoperative features, the performance matches that of all pre- and intraoperative features (see back box). (**A**) The predictive capability of preoperative neuropsychological tests was assessed for postoperative delirium (POD) individually and in combination with other features. (**B**) Our analysis, as depicted in the receiver operating characteristic curve, demonstrated the successful prediction of POD by preoperative neuropsychological tests. However, when combined with pre- and intraoperative data, the performance gains were only marginal. Asterisks (*) denote the Area Under the receiver operating characteristic Curve (AUC) values, which were assessed against chance permutation testing, revealing a significance (**P≤.001). (**C**) The AUC values of neuropsychological tests were compared to those integrated into the pre- and intraoperative features. Adding preoperative neuropsychological tests to the preoperative model improves POD prediction to a level comparable to the best-performing model, similar to the effect of incorporating intraoperative features. In contrast, using preoperative features alone without neuropsychological tests results in lower performance. (**D **) Specific neuropsychological tests, such as the Montreal Cognitive Assessment (MoCA) and Trail Making Tests (TMTs), were particularly influential in POD prediction.

### Ethical Considerations

This study was approved by the Ethics Commission of the Faculty of Medicine of the University of Tübingen (233/2017BO1, October 12, 2017) and the Ethics Commission of the University of Potsdam (38/2017, December 11, 2017), and was registered in the German Clinical Trials Register (DRKS00012797, July 2017).

All participants provided written informed consent for participation and for the publication of any potentially identifiable data. Data were pseudonymized, stored securely, and handled in accordance with applicable privacy and confidentiality regulations. Participants were not financially compensated but were reimbursed for travel expenses when applicable.

## Results

### Predicting POD With Combined, Pre-, and Intraoperative Features

The models incorporating combined and independent pre- and intraoperative features exhibited robust performance, as evidenced by AUC values surpassing chance levels, all with *P*<.002 (Table S4 in Multimedia Appendix 1) and Table S5 in Multimedia Appendix 1 displays the performance of these models through receiver operating characteristic curves with the random forest. The combined model using only preoperative features (Pre-Op) achieved an AUC of 0.760, comparable to a model incorporating both pre- and intraoperative features (Pre- and Intra-Op) with an AUC of 0.783, showing no statistically significant difference (Figure 1C and Table S6 in Multimedia Appendix 1). Independent models exclusively using preoperative clinical, preoperative surgical, or intraoperative surgical features also demonstrated high AUC values (0.691, 0.664, and 0.670, respectively) without statistically significant difference. Notably, key predictors of POD included surgery type and cardiopulmonary bypass (preoperative surgical; recruiting hospital and age (preoperative sociodemographic); ASA status, Clinical Frailty Scale, Barthel Index, polypharmacy, and creatinine clearance (preoperative clinical); cut-to-suture time, anesthesia duration, and blood loss (intraoperative surgical) shown in Figure 1D and Figure S2-A in Multimedia Appendix 1. Further details, including all pairwise model comparisons and *P* values for differences in AUC values, can be found in Table S6 in Multimedia Appendix 1. A detailed list of the feature importance can be found in Tables S7-S8 in Multimedia Appendix 1.

### Addition of Preoperative Neuropsychological Assessments

The model using preoperative neuropsychological assessments exclusively exhibited AUC values of 0.617. Integrating neuropsychological assessments into the model, using both pre- and intraoperative features (Pre- and Intra-Op+NeuroPsy), led to a slight improvement in the AUC, reaching 0.803 (Figure 2B and Table S6 in Multimedia Appendix 1). Adding neuropsychological assessments to the preoperative model (Pre-Op+ NeuroPsy) improved the AUC from 0.760 to 0.787, which resulted in a model that matched the above best-performing model (Figure 2C and Table S6 in Multimedia Appendix 1). Specifically, the MoCA scores and TMTs before surgery were important for predicting POD, as illustrated in Figure 2D and Figure S2-B in Multimedia Appendix 1. For detailed pairwise comparisons of AUC values and corresponding *P* values, please refer to Table S6 in Multimedia Appendix 1. A detailed list of the feature importance can be found in Tables S9-S10 in Multimedia Appendix 1.

### Robustness of Predictive Models in Performance Evaluation

Evaluating the performance of 4 classifiers with tuned hyperparameters revealed comparable results (Tables S12-S15 in Multimedia Appendix 1). The random forest model, demonstrating marginally better performance in AUC values in models with combined features, was highlighted above. In addition, random forest models demonstrated good calibration, as indicated by a consistent distribution of high-confidence predictions across different feature sets. The inclusion of intraoperative and neuropsychological information did not compromise calibration, ensuring the model’s reliability in distinguishing high- and low-risk patients (Figure S3 in Multimedia Appendix 1).

Including intervention allocation information had no discernible impact on predictive performance (Tables S5 and S16 in Multimedia Appendix 1). Finally, there was no statistically significant difference in AUC between models with and without oversampling (Table S17 in Multimedia Appendix 1).

## Discussion

### Principal Findings

Leveraging machine learning, we predicted the occurrence of POD after elective surgeries through a combination of preoperative and intraoperative features with a large multicenter cohort, achieving an AUC above 0.8. This performance exceeds that of traditional scoring systems such as the Delirium Risk Assessment Tool, Delirium Risk Assessment Score, and Delirium Elderly At-Risk, which have demonstrated AUC values between 0.5 and 0.7 in a large cohort [54-56]. Surgical information both before and during the surgery was critical in predicting POD. Integrating neuropsychological tests into preoperative features enhanced the AUC to a level comparable to the best-performing model, effectively replacing intraoperative features for predicting POD before surgery. This improvement was primarily driven by MoCA and TMTs A and B, as elucidated by model explanations. This integrated analysis improves conventional clinical risk profiling, furnishing superior predictive capacity with promising implications for surgical planning in the era of machine learning-assisted health care and empowering the prioritization of pivotal features in future work.

### Critical Surgical Information

Surgical features, both preoperative and intraoperative, emerged as good solo predictors category for POD (Figure 1C). While baseline clinical profiles, including ASA physical status, renal function, frailty, and polypharmacy, are important, intraoperative surgical features such as individual surgery and anesthesia duration and factors related to blood loss were particularly important for predicting POD (Figure 1D and Figure S2-A in Multimedia Appendix 1). In addition, preoperative surgical information such as types of surgery and use of cardiopulmonary bypass were also associated with a higher risk of POD, aligning with a higher overall incidence of cardiac surgery in patients with POD relative to those without POD (206/375, 54.9% vs 264/1249, 21.1% as indicated in Table S1 in Multimedia Appendix 1). Consistent with our findings, a 30-minute increase in surgery duration corresponded to a 6% rise in POD risk [28], and this risk is further elevated in prolonged cardiac surgeries using cardiopulmonary bypass [57], potentially leading to hypoperfusion or microembolism [58]. This suggests a potential cumulative effect, particularly in patients with poor preoperative clinical profiles. Overall, our findings highlight the critical importance of cardiovascular surgical risk measures in POD prediction.

Looking ahead, the integration of real-time predictive technology into surgical workflows holds promise. This advancement could potentially facilitate on-the-fly predictions during surgery, enabling timely adjustments to medication or nonpharmacological intervention to mitigate potential adverse outcomes associated with surgical interventions. Our study emphasizes the substantial value of information gleaned from measures taken during surgery, shedding light on their crucial role in enhancing our understanding and prediction of POD.

### Enhanced POD Prediction Before Elective Surgery Through Neuropsychological Assessments

With a larger cohort and a more comprehensive battery of preoperative neuropsychological assessments, this study demonstrated slightly improved predictive performance compared to a previous model that used a smaller sample and a limited neuropsychological assessment (MoCA) [37]. Consistent with prior research [16], our results indicate that preoperative models can achieve comparable performance to those incorporating intraoperative features in predicting POD. Although incorporating intraoperative data slightly improved POD predictions, they do not diminish the value of neuropsychological testing for early risk stratification, especially in elective surgeries where timing allows for actionable planning. A key limitation of intraoperative data prevents surgical planning and decision-making beforehand, allowing only adjustments in real time. These findings are particularly relevant to older patients undergoing elective surgery, as they have sufficient preoperative time for noninvasive neuropsychological assessments and for adjusting surgical strategies accordingly. Therefore, augmenting the predictive performance by incorporating data that can be gathered before a surgical procedure is important, as it allows for potential prehabilitation strategies, integrated surgical planning, and informed decision making prior to any invasive or surgical procedure.

In our study, preoperative neuropsychological assessments were predictive above chance (Figure 2B). To achieve performance comparable to the model combining all available features, adding neuropsychological tests to the preoperative model can effectively replace intraoperative features to predict POD before surgery (Figure 2C), which is critical given that surgical and postoperative management could be optimized. Preoperative models with neuropsychological tests effectively predicted POD before surgery substantiating previous observations [13,29,59]. This could be attributed to preoperative neuropsychological tests revealing subtle cognitive deficits that are not captured by dementia or delirium history. Fewer than 2% of patients in this cohort reported a diagnosis of mild or moderate dementia before elective surgery (Table S1 in Multimedia Appendix 1), and neither dementia nor delirium history was influential predictors with consistently almost zero feature attributions (Tables S7-S10 in Multimedia Appendix 1). These subtle deficits may progress into POD. In line with this explanation, timely preoperative cognitive interventions can mitigate the risk of POD and long-term cognitive dysfunction after cardiac surgeries [60,61]. In addition, patients with pre-existing cognitive decline face increased risks of other postoperative complications [62,63]. Consequently, baseline neuropsychological assessments are valuable for improving the prediction of POD beyond what clinical history alone can offer.

Selecting suitable neuropsychological assessments for clinical use is crucial. Our study identified the MoCA and TMTs as effectively indicated by their average absolute SHAP values (Figure 2D and Figure S2-B in Multimedia Appendix 1). Low scores on the MoCA and longer test times on the TMT indicate poor cognitive performance and executive dysfunction. These tests are crucial for predicting POD risk, as demonstrated in a previous prediction study [15]. Our findings, while focused on prediction [64], can complement previous etiological studies that show patients with mild cognitive impairment at baseline are more likely to develop POD [26], while good preoperative cognitive performance is protective against POD [18]. Previous studies have often used the MMSE [29,32,65], Clock-Drawing Test [20], or MoCA score as preoperative risk factors [66]. Critically, we replicated the strong association between baseline MoCA and POD risk in the previous theory-driven etiological PAWEL-R study with a larger and extended cohort [13]. These findings offer a thorough understanding of the efficacy of individual preoperative neuropsychological tests in predicting POD. By conducting comprehensive assessments of predictors for pre-existing risks, we may unlock new avenues for optimizing surgical planning and postoperative management. Our study underscores the added, albeit moderate, advantage of evaluating cognition, emphasizing its importance and advocating for its inclusion in future developments aimed at refining preoperative risk assessments.

### Limitations and Recommendations

The study exhibits several limitations that require consideration. First, interpreting and using SHAP values warrants caution [67], as is generally the case for methods using model explanations in medicine [68,69]. Unstable explanations are not uncommon for complex models trained on large datasets [70,71]. While the ranking of importance may fluctuate, features with higher mean absolute SHAP values generally maintain consistent attributions. To enhance stability and reduce dependence on specific cohort segments, we aggregated SHAP values across multiple folds with shuffling, ensuring a more reliable assessment of feature importance (Tables S7-S10 in Multimedia Appendix 1). Second, although permutation is a robust statistical method, its conservative nature means nonsignificance does not always indicate the absence of a difference. Third, to minimize temporal bias and avoid causal leakage, we excluded perioperative features whose timing could not be clearly verified to precede the onset of delirium. While such features may have clinical relevance, their inclusion risks introducing postoutcome information. We recommend future research explore causal-learning approaches to better define temporal relationships, though limitations of observational data must be considered [72]. Fourth, we harnessed cognitive data derived from standardized assessments. However, it is crucial to note that these procedures, while standardized, often remain nondigitalized. This presents significant untapped potential for future advancements in the realm of risk prediction before surgical procedures. Fifth, while our study included a wide range of preoperative neuropsychological assessments, it is not an exhaustive list. As neuropsychological assessments are time-consuming and require training for assessors to conduct accurate tests and interpret results. Therefore, incorporating semiautomatic assessments of cognition [73] may prove advantageous and a relevant direction for future research.

### Conclusion and Relevance

This study highlights the feasibility of predicting POD before elective surgery in adults aged 70 years and older by using a diverse set of features, including neuropsychological assessments. Our research advances the understanding of POD predictors, enabling a more targeted approach to POD risk prediction in clinical practice. The findings offer crucial insights into predictive features for POD, underscoring the importance of integrating these predictors into the digital transformation of preoperative risk assessment.

## Supplementary material

10.2196/67958 Multimedia Appendix 1 Additional figures and tables.

10.2196/67958 Checklist 1 CONSORT-EHEALTH (Consolidated Standards of Reporting Trials of Electronic and Mobile Health Applications and Online Telehealth; version 1.6.1) checklist.

## Abbreviations

ASA: American Society of Anesthesiologists
AUC: area under the receiver operating characteristic curve
CONSORT-EHEALTH: Consolidated Standards of Reporting Trials of Electronic and Mobile Health Applications and Online Telehealth
CRP: C-reactive protein
I-CAM: Confusion Assessment Method
MMSE: Mini-Mental State Examination
MoCA: Montreal Cognitive Assessment
PAWEL: Patientensicherheit, Wirtschaftlichkeit und Lebensqualität bei elektiven Operationen
PAWEL-R: Patientensicherheit, Wirtschaftlichkeit und Lebensqualität bei elektiven Operationen–Risk
PHQ-4: Patient Health Questionnaire-4
POD: postoperative delirium
SHAP: Shapley Additive Explanations
SMI: Subjective Memory Impairment
STROBE: Strengthening the Reporting of Observational studies in Epidemiology
TMT: Trail Making Test
