# Supplementary material for: Predicting Postoperative Delirium in Older Patients Before Elective Surgery: Multicenter Retrospective Cohort Study
Source: JMIR Aging. 2025 Aug 19;8:e67958. doi: 10.2196/67958 (PMC12364014; doi:10.2196/67958)
Supplement: Multimedia Appendix 1 [file aging-v8-e67958-s001.docx]

**Predicting Postoperative Delirium in Older** **Patients before Elective Surgery: a multicenter retrospective cohort study**

Shun-Chin Jim Wu, MD, MSc1,2,3*, Nitin Sharma, MSc1,2*, Anne Bauch, PhD1,2, Hao-Chun Yang, PhD1,2, Jasmine L. Hect, BSc4, Christine Thomas, MD1,5, Sören Wagner, MD6,7, Bernd R. Förstner, PhD8, Christine A.F. von Arnim, MD9,10, Tobias Kaufmann, PhD1,2,11, Gerhard W. Eschweiler, MD1,2,12, Thomas Wolfers, PhD1,2,11,13, for the PAWEL Study Group

* Equal contribution

**Supplementary Figures and Tables**

| **Figure S1.** Flow diagram of patients whose data were received and excluded for the study |
| --- |
| 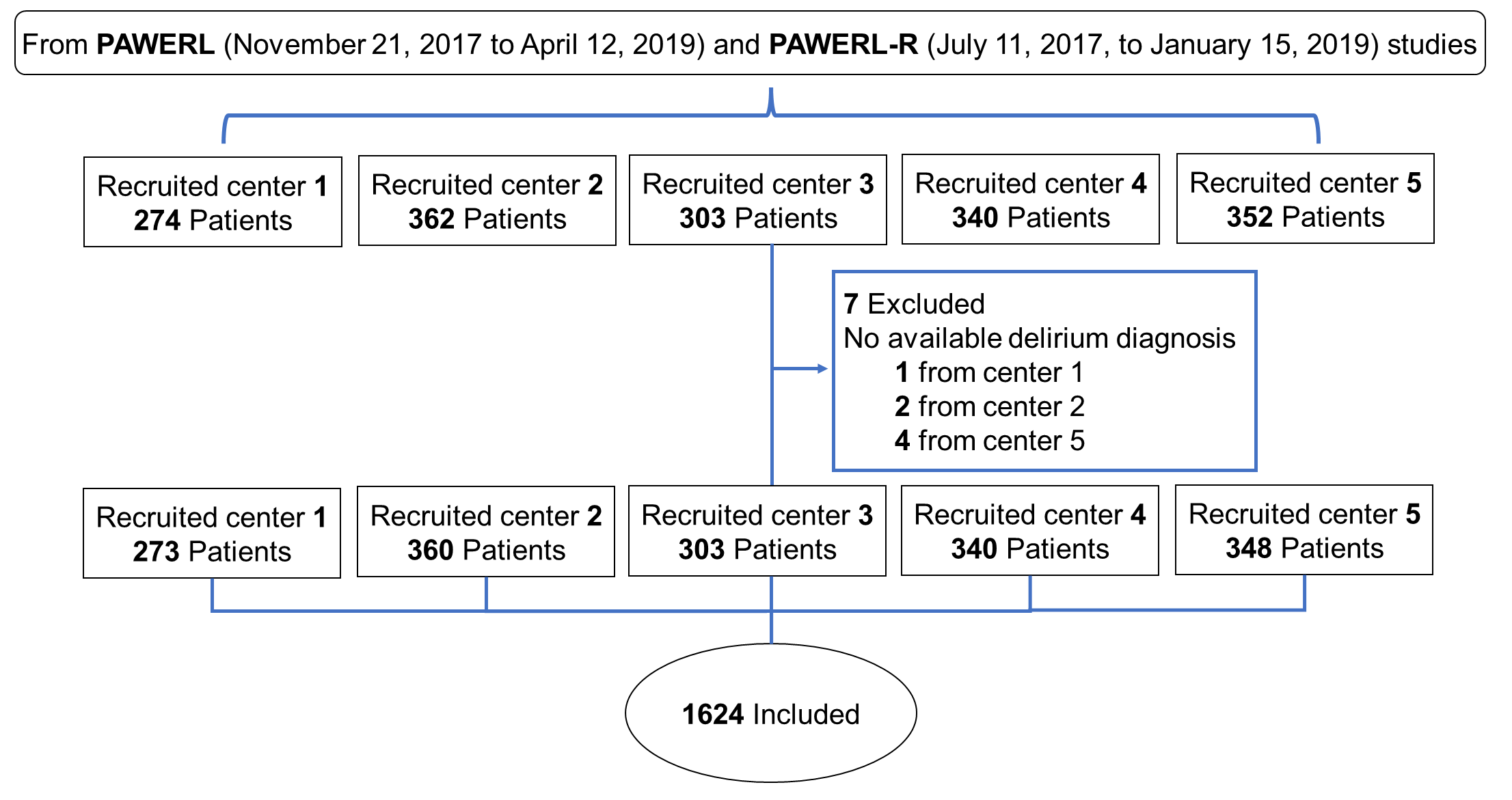 |

**Figure S2.** Feature attribution for top 15 features in models with Preoperative: Sociodemographic, Clinical, Surgical; Preoperative: Sociodemographic, Clinical, Surgical, Neuropsychological and Intraoperative: Clinical, Surgical

| 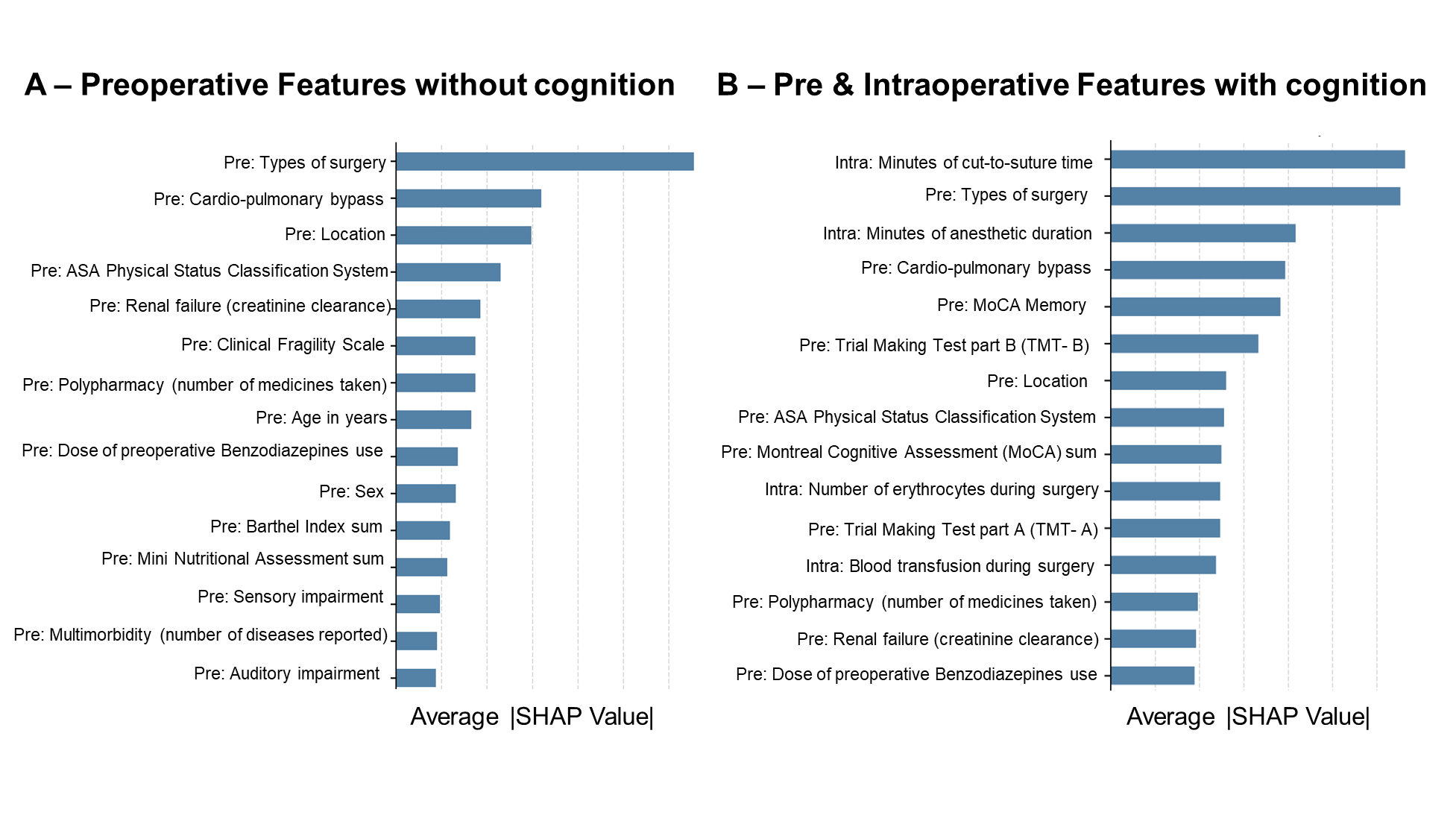 |
| --- |
| (A) Preoperative feature attribution based on averaged absolute SHapley Additive exPlanations (SHAP) values aggregated across folds show that preoperative surgical information such as types of surgery and cardio-pulmonary bypass were critical for predicting postoperative delirium (POD). (B) Intraoperative surgical information such as surgical and anaesthesia duration and preoperative neuropsychological tests such as Montreal Cognitive Assessment (MoCA) and longer Trail Making Test (TMT) were also essential for POD prediction. |

**Figure S3.** Calibration Plots (Original and after Platting Scaling) and High-Confidence Predictions (after Platting Scaling) for Random Forest Models

| Model | Positive Predictions ( > 0.9) | Negative Predictions ( < 0.1) | Calibration Plot |
| --- | --- | --- | --- |
| Preoperative: Sociodemographic, Clinical, Surgical  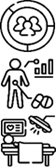 | *23.20 %* | *83.03 %* | *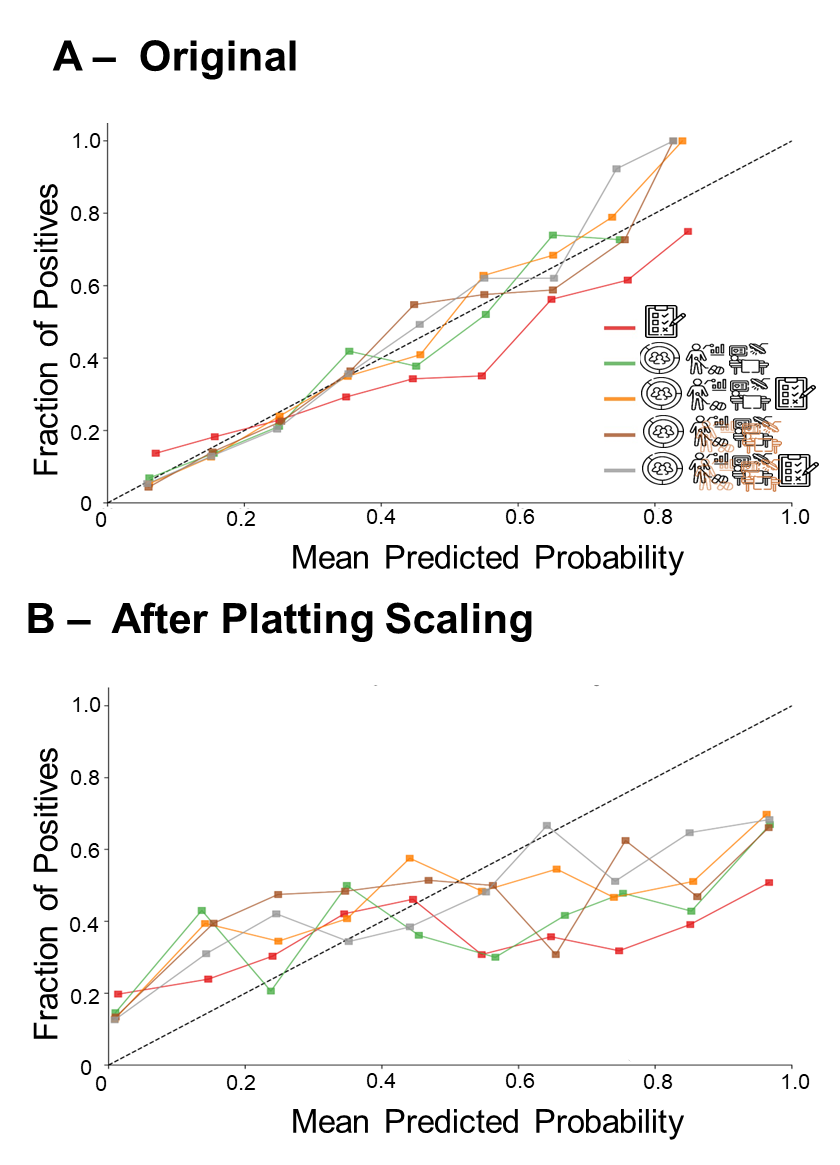* |
| Preoperative: Sociodemographic, Clinical, Surgical and Intraoperative : Clinical, Surgical  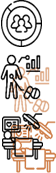 | *20.80 %* | *84.31 %* |  |
| Preoperative: Neuropsychological  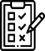 | *8.53 %* | *82.71 %* |  |
| Preoperative: Sociodemographic, Clinical, Surgical, Neuropsychological  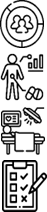 | ***23.47 %*** | ***84.47 %*** |  |
| Preoperative: Sociodemographic, Clinical, Surgical, Neuropsychological and Intraoperative: Clinical, Surgical  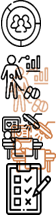 | *21.87 %* | *83.43 %* |  |

Random forest models demonstrate good calibration across different feature sets. Notably, combined preoperative features, including neuropsychological assessments, yielded the highest positive prediction rates (>0.9) at 23.47% and the highest negative prediction rates (<0.1) at 84.47% (**bold**).

**Table S1.** Patient characteristics and group comparison

| Sociodemographic (preoperative) | Delirium (N=375) | Non-Delirium (N=1249) | *P* | |
| --- | --- | --- | --- | --- |
| Sex ratio (M:F) | 237:138 | 613:636 | <0.001** | |
| Age in years (SD) ^+^ | 78.34 (4.97) | 77.74 (4.86) | 0.195 | |
| Number of participants in each location | 38:74:47:102:114 | 235:286:256:238:234 | <0.001** | |
| Years of education in total (SD) | 12.6 (2.87) | 12.84 (2.9) | 0.134 | |
| Alcohol consumption per month (SD) | 0.03 (.18) | 0.02 (.15) | 0.430 | |
| Cigarette consumption per day (SD)  ^+^ | 0.59 (2.67) | 0.58 (2.89) | 0.874 | |
| Alone living (%) | 113 (30.1%) | 362 (29.0%) | 0.715 | |
| Received non-pharmacological interventions for delirium (%) | 163 (43.5%) | 606 (48.5%) | 0.097 | |
| Cognitive information and psychological screens (preoperative) | **Delirium (N=375)** | **Non-Delirium (N=1249)** | ***P*** | |
| Montreal Cognitive Assessment (MoCA) sum score at admission (SD) | 20.97 (4.63) | 22.99 (3.41) | <0.001** | |
| MoCA Attention at admission (SD) | 4.94 (1.31) | 5.25 (0.99) | <0.001** | |
| MoCA Memory at admission (SD) | 1.74 (1.66) | 2.55 (1.65) | <0.001** | |
| MoCA Orientation at admission (SD) | 5.60 (0.97) | 5.89 (0.41) | <0.001** | |
| MoCA Visuospatial/Executive at admission (SD) | 2.91 (1.44) | 3.21 (1.31) | <0.001** | |
| MoCA Naming at admission (SD) | 2.86 (0.48) | 2.92 (0.32) | 0.025* | |
| MoCA Language at admission (SD) | 1.57 (0.94) | 1.84 (0.91) | <0.001** | |
| MoCA Abstraction at admission (SD) | 1.44 (0.69) | 1.53 (0.67) | 0.034* | |
| Trial Making Test part A (TMT-A) in seconds at admission (SD) | 74.01 (39.5) | 62.33 (28.2) | <0.001** | |
| Trial Making Test part B (TMT-B) in seconds at admission (SD) | 183.6 (75.2) | 154.3 (68.2) | <0.001** | |
| Digit span (length) at admission (SD) | 3.71 (1.29) | 4.0 (1.19) | <0.001** | |
| Patient Health Questionnaire-4 (PHQ-4) at admission (median, SD)^+^ | 1 (2.78) | 1 (2.61) | 0.718 | |
| PHQ-4 anxiety rating at admission (median, SD)^+^ | 1 (1.49) | 1 (1.39) | 0.417 | |
| PHQ-4 depression rating at admission (median, SD)^+^ | 1 (1.66) | 1 (1.58) | 0.289 | |
| Subjective memory impairment (SMI) at admission (median, SD)^+^ | 1 (0.55) | 1 (0.53) | 0.121 | |
| Clinical information (preoperative and intraoperative) | **Delirium (N=375)** | **Non-Delirium (N=1249)** | ***P*** | |
| Preoperative: Hemoglobin level (g/dl) at admission (SD) | 13.42 (6.53) | 13.65 (7.33) | 0.572 | |
| Preoperative: Sodium level (mmol/l) at admission (SD) | 140.1 (2.86) | 139.9 (3.00) | 0.354 | |
| Preoperative: C-reactive protein (mg/l) at admission (SD) | 9.21 (18.98) | 8.44 (19.97) | 0.530 | |
| Preoperative: Renal failure (creatinine clearance in mg/dl) at admission (SD) | 64.99 (24.4) | 69.00 (30.7) | 0.010* | |
| Preoperative: Polypharmacy (number of medicines taken) at admission (SD) ^+^ | 7.28 (3.71) | 6.03 (3.65) | 0.012* | |
| Preoperative: Dose (mg) of preoperative Benzodiazepines use (SD) ^+^ | 7.90 (14.42) | 3.55 (13.24) | <0.001** | |
| Preoperative: Dose (mg) of preoperative Neuroleptics use (SD) ^+^ | 1.05 (10.3) | 0.14 (2.47) | 0.617 | |
| Preoperative: Dose (mg) of preoperative Opiates use (SD) | 0.83 (11.57) | 2.33 (21.3) | 0.081 | |
| Preoperative: Dose (mg) of preoperative Propofol use (SD) | 2.57 (19.3) | 9.78 (105.5) | 0.023* | |
| Preoperative: Mini Nutritional Assessment (MNA) sum score at admission (SD) | 11.08 (2.71) | 11.43 (2.58) | 0.026* | |
| Preoperative: Multimorbidity (number of diseases reported) at admission (SD) ^+^ | 0.96 (0.66) | 0.78 (0.67) | 0.113 | |
| Preoperative: ASA Physical Status Classification System (median) at admission (SD) | 3 (0.60) | 3 (0.63) | <0.001** | |
| Preoperative: Charlson Comorbidity Index at admission (SD) ^+^ | 2.53 (2.23) | 2.21 (2.25) | 0.255 | |
| Preoperative: Barthel Index sum score at admission (SD) | 91.08 (19.4) | 93.66 (14.6) | 0.02* | |
| Preoperative: Clinical Frailty Scale (median) at admission (SD) | 3 (1.41) | 3 (1.33) | 0.005* | |
| Preoperative: Auditory impairment at admission (%) | 251 (68.6%) | 674 (56.5%) | 0.027* | |
| Preoperative: Visual impairment at admission (%) | 74 (20.6%) | 229 (19.7%) | 0.764 | |
| Preoperative: Any sensory impairment at admission (%) | 233 (65.3%) | 650 (56.3%) | 0.025* | |
| Preoperative: Preexisting dementia (%) | 22 (5.9%) | 7 (0.6%) | <0.001** | |
| Preoperative: Previous delirium history (%) | 38 (10.4%) | 100 (8.2%) | .225 | |
| Intraoperative: Dose (mg) of intraoperative Benzodiazepines use (SD) ^+^ | 1.75 (4.85) | 1.41 (14.21) | 0.952 |  |
| Intraoperative: Dose (mg) of intraoperative Neuroleptics use (SD) ^+^ | 0.16 (2.02) | 0.08 (2.14) | 0.617 |  |
| Intraoperative: Dose (mg) of intraoperative Opiates use (SD) | 9.65 (81.3) | 10.82 (208) | 0.872 |  |
| Intraoperative: Dose (mg) of intraoperative Propofol use (SD) | 522 (675.5) | 297 (471.8) | <0.001** |  |
| Surgery (preoperative and intraoperative) | **Delirium (N=375)** | **Non-Delirium (N=1249)** | ***P*** | |
| Preoperative: Types of surgery  (Joint: Spine: Vessels: Heart: Lung: Abdominal: Urogenital: Others) | 100:11:22:206:1:21:7:7 | 574:100:126:264:1:108:49:27 | <0.001** | |
| Preoperative: Types of anesthesia (Intubation: Laryngeal: Spinal) | 318:14:31 | 907:78:207 | <0.001** | |
| Preoperative: Inhalation anesthesia (%) | 284 (75.7%) | 751 (60.1%) | <0.001** | |
| Preoperative: Regional anesthesia (%) | 87 (23.2%) | 469 (37.6%) | <0.001** | |
| Preoperative: Cardio-pulmonary bypass with heart-lung machine use (%) | 176 (47.1%) | 220 (17.8%) | <0.001** | |
| Intraoperative: Minutes of cut-to-suture time (SD) ^+^ | 191.3 (95.5) | 131.5 (75.4) | <0.001** | |
| Intraoperative: Minutes of anesthetic duration (SD) | 252.9 (104) | 201.0 (84.5) | <0.001** |  |
| Intraoperative: Number of erythrocytes during surgery (SD) | .91 (1.68) | .28 (.90) | <0.001** |  |
| Intraoperative: Blood pressure drop during surgery (%) | 125 (33.6%) | 488 (39.5%) | 0.046* |  |
| Intraoperative: Blood transfusion during surgery (%) | 122 (32.8%) | 133 (10.7%) | <0.001** | |
| Intraoperative: Drop in hemoglobin value >3g/dl during surgery (%) | 196 (53.3%) | 449 (37.5%) | <0.001** | |
| Intraoperative: Increase in creatine >0.3mg/dl during surgery (%) | 49 (13.5%) | 59 (4.8%) | <0.001** | |

^+^: nonparametric Mann-Whitney U tests for continuous/discrete variables without normality or ordinal variables; **P* <.05, ***P* ≤.001.

The range of possible scores for the Montreal Cognitive Assessment (MoCA) are as follows: MoCA sum score (0-30), MoCA Attention (0-6), MoCA Memory (0-5), MoCA Orientation (0-6), MoCA Visuospatial/Executive (0-5), MoCA Naming (0-3), MoCA Language (0-3), and MoCA Abstraction (0-2).

**Table S2.** Patient outcomes after surgery and group comparison

| Outcome | Delirium (N=375) | Non-Delirium (N=1249) | *P* |
| --- | --- | --- | --- |
| Attention deficit in CAM at discharge (%) | 42 (11.4%) | 52 (4.5%) | <0.001** |
| Disorganized thinking rating in CAM at discharge (SD) | 0 (0.49) | 0 (0.38) | 0.005* |
| Hyperactivity in CAM at discharge (%) | 4 (1.1%) | 2 (0.2%) | 0.040* |
| Psychomotor behavior in CAM at discharge (%) | 7 (1.9%) | 11 (0.89%) | 0.120 |
| Drowsiness in CAM at discharge (%) | 5 (1.4%) | 3 (0.2%) | 0.025* |
| Barthel Index sum score at discharge (SD) | 74.82 (26.3) | 88.4 (15.6) | <0.001** |
| MoCA sum score at discharge (SD) | 20.01 (4.69) | 22.65 (3.57) | <0.001** |
| MoCA Attention at discharge (SD) | 4.67 (1.45) | 5.17 (1.05) | <0.001** |
| MoCA Memory at discharge (SD) | 1.62 (1.58) | 2.35 (1.63) | <0.001** |
| MoCA Orientation at discharge (SD) | 5.42 (1.06) | 5.8 (0.54) | <0.001** |
| MoCA Visuospatial/Executive at discharge (SD) | 2.89 (1.45) | 3.37 (1.30) | <0.001** |
| MoCA Naming at discharge (SD) | 2.58 (0.64) | 2.72 (0.55) | <0.001** |
| MoCA Language at discharge (SD) | 1.14 (0.98) | 1.62 (1.04) | <0.001** |
| MoCA Abstraction at discharge (SD) | 1.72 (0.57) | 1.86 (0.42) | <0.001** |
| TMT-A at discharge (SD) | 81.39 (43.0) | 64.27 (31.7) | <0.001** |
| TMT-B at discharge (SD) | 198.5 (77.2) | 158.0 (71.4) | <0.001** |
| Digit span (length) at discharge (SD) | 3.49 (1.33) | 3.96 (1.20) | <0.001** |
| Days in Intermediate Care Unit stay after surgery (SD) ^+^ | 0.77 (1.52) | 0.39 (0.91) | 0.637 |
| Days of Intensive Care Unit stay after surgery (SD) ^+^ | 2.60 (2.70) | .68 (1.19) | <0.001** |
| Days of hospitalization (SD) ^+^ | 8.66 (2.06) | 8.02 (2.31) | <0.001** |

^+^: nonparametric Mann-Whitney U tests for continuous/discrete variables without normality or ordinal variables; *: P<.05; **: P<.001

**Table S3**. Information about missing values in data and outcome

| Sociodemographic (preoperative) | Delirium (N=375) | Non-Delirium (N=1249) | All subjects (N=1624) |
| --- | --- | --- | --- |
| Sex (%) | 0 (0%) | 0 (0%) | 0 (0%) |
| Age in years (%) | 0 (0%) | 0 (0%) | 0 (0%) |
| Location (%) | 0 (0%) | 0 (0%) | 0 (0%) |
| Years of education in total (%) | 2 (0.5%) | 3 (0.2%) | 5 (0.3%) |
| Alcohol consumption per month (%) | 2 (0.5%) | 8 (0.6%) | 10 (0.6%) |
| Cigarette consumption per day (%) | 0 (0%) | 0 (0%) | 0 (0%) |
| Alone living (%) | 0 (0%) | 0 (0%) | 0 (0%) |
| Received non-pharmacological interventions for delirium (%) | 0 (0%) | 0 (0%) | 0 (0%) |
| Cognitive information and psychological screens (preoperative) | **Delirium (N=375)** | **Non-Delirium (N=1249)** | **All subjects (N=1624)** |
| Montreal Cognitive Assessment (MoCA) sum score at admission (%) | 14 (3.7%) | 34 (2.7%) | 48 (3.0%) |
| MoCA Attention at admission (%) | 5 (1.3%) | 12 (1.0%) | 17 (1.1%) |
| MoCA Memory at admission (%) | 5 (1.3%) | 14 (1.1%) | 19 (1.2%) |
| MoCA Orientation at admission (%) | 5 (1.3%) | 11 (0.9%) | 16 (1.0%) |
| MoCA Visuospatial/Executive at admission (%) | 12 (3.2%) | 29 (2.3%) | 41 (2.5%) |
| MoCA Naming at admission (%) | 4 (1.1%) | 9 (0.7%) | 13 (0.8%) |
| MoCA Language at admission (%) | 5 (1.3%) | 13 (1.0%) | 18 (1.1%) |
| MoCA Abstraction at admission (%) | 5 (1.3%) | 14 (1.1%) | 19 (1.2%) |
| Trial Making Test part A (TMT-A) in seconds at admission (%) | 53 (14.1%) | 158 (12.7%) | 211 (13.0%) |
| Trial Making Test part B (TMT-B) in seconds at admission (%) | 81 (21.6%) | 223 (17.9%) | 304 (18.7%) |
| Digit span (length) at admission (%) | 56 (14.9%) | 152 (12.2%) | 208 (12.8%) |
| Patient Health Questionnaire-4 (PHQ-4) at admission (%) | 17 (4.5%) | 57 (4.6%) | 74 (4.6%) |
| PHQ-4 anxiety rating at admission (%) | 17 (4.5%) | 53 (4.2%) | 70 (4.3%) |
| PHQ-4 depression rating at admission (%) | 17 (4.5%) | 56 (4.5%) | 73 (4.5%) |
| Subjective memory impairment (SMI) at admission (%) | 1 (0.3%) | 4 (0.3%) | 5 (0.3%) |
| Clinical information (preoperative and intraoperative) | **Delirium (N=375)** | **Non-Delirium (N=1249)** | **All subjects (N=1624)** |
| Preoperative: Hemoglobin level (g/dl) at admission (%) | 6 (1.6%) | 30 (2.4%) | 36 (2.2%) |
| Preoperative: Sodium level (mmol/l) at admission (%) | 8 (2.1%) | 36 (2.9%) | 44 (2.7%) |
| Preoperative: C-reactive protein (mg/l) at admission (%) | 53 (14.1%) | 246 (19.7%) | 299 (18.4%) |
| Preoperative: Renal failure (creatinine clearance in mg/dl) at admission (%) | 11 (2.9%) | 43 (3.4%) | 54 (3.3%) |
| Preoperative: Polypharmacy (number of medicines taken) at admission (%) | 0 (0%) | 0 (0%) | 0 (0%) |
| Preoperative: Dose (mg) of preoperative Benzodiazepines use (%) | 0 (0%) | 3 (0.2%) | 3 (0.2%) |
| Preoperative: Dose (mg) of preoperative Neuroleptics use (%) | 0 (0%) | 4 (0.3%) | 4 (0.3%) |
| Preoperative: Dose (mg) of preoperative Opiates use (%) | 2 (0.5%) | 24 (1.9%) | 26 (1.6%) |
| Preoperative: Dose (mg) of preoperative Propofol use (%) | 5 (1.3%) | 18 (1.4%) | 23 (1.4%) |
| Preoperative: Mini Nutritional Assessment (MNA) sum score at admission (%) | 9 (2.4%) | 31 (2.5%) | 40 (2.5%) |
| Preoperative: Multimorbidity ( number of diseases reported) at admission (%) | 0 (0%) | 0 (0%) | 0 (0%) |
| Preoperative: ASA Physical Status Classification System (median) at admission (%) | 7 (1.9%) | 33 (2.6%) | 40 (2.5%) |
| Preoperative: Charlson Comorbidity Index at admission (%) | 0 (0%) | 0 (0%) | 0 (0%) |
| Preoperative: Barthel Index sum score at admission (%) | 10 (2.7%) | 37 (3.0%) | 47 (2.9%) |
| Preoperative: Clinical Frailty Scale (median) at admission (%) | 2 (0.5%) | 19 (1.5%) | 21 (1.3%) |
| Preoperative: Auditory impairment at admission (%) | 9 (2.4%) | 57 (4.6%) | 66 (4.1%) |
| Preoperative: Visual impairment at admission (%) | 16 (4.3%) | 87 (7.0%) | 103 (6.3%) |
| Preoperative: Sensory impairment at admission (%) | 18 (4.8%) | 95 (7.6%) | 113 (7.0%) |
| Preoperative: Preexisting dementia (%) | 0 (0%) | 0 (0%) | 0 (0%) |
| Preoperative: Previous delirium history (%) | 9 (2.4%) | 25 (2%) | 34 (2.1%) |
| Intraoperative: Dose (mg) of intraoperative Benzodiazepines use (%) | 0 (0%) | 3 (0.2%) | 3 (0.2%) |
| Intraoperative: Dose (mg) of intraoperative Neuroleptics use (%) | 0 (0%) | 4 (0.3%) | 4 (0.3%) |
| Intraoperative: Dose (mg) of intraoperative Opiates use (%) | 2 (0.5%) | 19 (1.5%) | 21 (1.3%) |
| Intraoperative: Dose (mg) of intraoperative Propofol use (%) | 2 (0.5%) | 5 (0.4%) | 7 (0.4%) |
| Surgery (preoperative and intraoperative) | **Delirium (N=375)** | **Non-Delirium (N=1249)** | **All subjects (N=1624)** |
| Preoperative: Types of surgery (%) | 0 (0%) | 0 (0%) | 0 (0%) |
| Preoperative: Types of anesthesia (%) | 12 (3.2%) | 57 (4.6%) | 69 (4.3%) |
| Preoperative: Inhalation anesthesia (%) | 0 (0%) | 0 (0%) | 0 (0%) |
| Preoperative: Regional anesthesia (%) | 0 (0%) | 0 (0%) | 0 (0%) |
| Preoperative: Preoperative: Cardio-pulmonary bypass with heart-lung machine use (%) | 1 (0.3%) | 11 (0.9%) | 12 (0.7%) |
| Intraoperative: Minutes of cut-to-suture time (%) | 1 (.27%) | 0 (0%) | 1 (0.1%) |
| Intraoperative: Minutes of anesthetic duration (%) | 79 (21.0%) | 126 (10.1%) | 205 (12.6%) |
| Intraoperative: Number of erythrocytes during surgery (%) | 26 (6.9%) | 127 (10.2%) | 153 (9.4%) |
| Intraoperative: Blood pressure drops during surgery (%) | 3 (0.8%) | 14 (1.1%) | 17 (1.1%) |
| Intraoperative: Blood transfusion during surgery (%) | 3 (0.8%) | 10 (0.8%) | 13 (0.8%) |
| Intraoperative: Drop in hemoglobin value >3g/dl during surgery (%) | 7 (1.9%) | 50 (4%) | 57 (3.5%) |
| Intraoperative: Increase in creatine >0.3mg/dl during surgery (%) | 12 (3.2%) | 77 (6.2%) | 89 (5.5%) |

**Table S4.** Model performance in area under the receiver operating characteristic curve compared to random chance (null hypothesis) : 1000 permutation tests.

| Model | *P* |
| --- | --- |
| Preoperative: Sociodemographic | 0.002 |
| Preoperative: Clinical | 0.001 |
| Preoperative: Surgical | 0.001 |
| Intraoperative : Clinical | 0.001 |
| Intraoperative : Surgical | 0.001 |
| Preoperative: Sociodemographic, Clinical, Surgical | 0.001 |
| Preoperative: Sociodemographic, Clinical, Surgical and Intraoperative : Clinical, Surgical | 0.001 |
| Preoperative: Neuropsychological | 0.001 |
| Preoperative: Sociodemographic, Clinical, Surgical, Neuropsychological | 0.001 |
| Preoperative: Sociodemographic, Clinical, Surgical, Neuropsychological and Intraoperative: Clinical, Surgical | 0.001 |

**Table S5.** Performance across all feature combinations with the random forest

| Model: mean (95% bootstrapped confidence interval) | ROC | PRC | Sen | Spe | Prec | Rec | BA |
| --- | --- | --- | --- | --- | --- | --- | --- |
| Preoperative: Sociodemographic | 0.591 (0.572-0.609) | 0.290 (0.273-0.309) | 20.115 (17.070-23.200) | 85.935 (84.470-87.430) | 30.045 (26.558-33.912) | 20.115 (17.070-23.200) | 53.024 (51.500-54.610) |
| Preoperative: Clinical | 0.691 (0.676-0.705) | 0.405 (0.385-0.428) | 17.210 (14.670-19.730) | 95.677 (94.960-96.482) | 54.460 (49.179-60.004) | 17.210 (14.670-19.730) | 56.442 (55.210-57.660) |
| Preoperative: Surgical | 0.664 (0.651-0.677) | 0.353 (0.339-0.366) | 0.767 (0.000-1.600) | 99.193 (98.320-99.600) | 22.605 (0.000-42.860) | 0.767 (0.000-1.600) | 49.980 (49.550-50.390) |
| Intraoperative : Clinical | 0.576 (0.555-0.593) | 0.337 (0.319-0.358) | 22.789 (20.263-25.070) | 89.235 (87.910-90.470) | 38.895 (35.020-42.520) | 22.789 (20.263-25.070) | 56.014 (54.610-57.300) |
| Intraoperative : Surgical | 0.670 (0.653-0.686) | 0.388 (0.364-0.409) | 28.687 (25.600-31.730) | 89.824 (88.630-91.030) | 45.860 (42.039-49.570) | 28.687 (25.600-31.730) | 59.257 (57.680-60.780) |
| Preoperative: Sociodemographic, Clinical, Surgical | 0.760 (0.749-0.771) | 0.496 (0.474-0.517) | 26.343 (24.000-28.537) | 94.645 (93.918-95.362) | 59.631 (55.618-63.460) | 26.343 (24.000-28.537) | 60.491 (59.289-61.640) |
| Preoperative: Sociodemographic, Clinical, Surgical and  Intraoperative: Clinical, Surgical | 0.783 (0.772-0.792) | 0.519 (0.499-0.538) | 27.536 (25.070-29.876) | 94.886 (94.160-95.602) | 61.782 (57.580-65.703) | 27.536 (25.070-29.876) | 61.208 (59.840-62.490) |
| Preoperative: Neuropsychological | 0.617 (0.601-0.634) | 0.367 (0.349-0.385) | 12.462 (10.670-14.130) | 96.387 (95.680-97.120) | 50.919 (44.680-57.140) | 12.462 (10.670-14.130) | 54.424 (53.440-55.400) |
| Preoperative: Sociodemographic, Clinical, Surgical, Neuropsychological | 0.787 (0.777-0.796) | 0.538 (0.518-0.558) | 31.777 (29.070-34.400) | 94.685 (93.920-95.440) | 64.225 (60.220-67.802) | 31.777 (29.070-34.400) | 63.228 (61.779-64.611) |
| Preoperative: Sociodemographic, Clinical, Surgical, Neuropsychological, Intervention allocation | 0.787 (0.778-0.797) | 0.538 (0.519-0.559) | 31.569 (28.800-34.130) | 94.770 (94.000-95.520) | 64.439 (60.748-68.213) | 31.569 (28.800-34.130) | 63.166 (61.810-64.540) |
| Preoperative: Sociodemographic, Clinical, Surgical, Neuropsychological and Intraoperative: Clinical, Surgical | 0.803 (0.794-0.811) | 0.555 (0.533-0.577) | 32.294 (29.330-35.200) | 94.869 (94.160-95.520) | 65.382 (61.410-69.100) | 32.294 (29.330-35.200) | 63.578 (62.160-65.020) |
| Preoperative: Sociodemographic, Clinical, Surgical, Neuropsychological, Intervention allocation and Intraoperative: Clinical, Surgical | 0.803 (0.796-0.812) | 0.555 (0.536-0.574) | 32.106 (29.600-34.670) | 94.900 (94.160-95.600) | 65.394 (61.689-68.892) | 32.106 (29.600-34.670) | 63.500 (62.140-64.811) |

ROC = area under the receiver operating characteristic curve; PRC= area under the precision-recall curve; Sen= sensitivity; Spe=specificity; Prec= precision; Rec= recall; BA= balanced accuracy

**Table S6.** Model performance difference in area under the receiver operating characteristic curve: 1000 permutation tests.

| *P* | 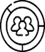 | 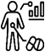 | 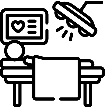 | 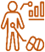 | 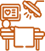 | 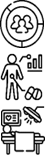 | 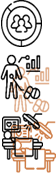 | 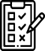 | 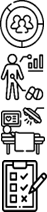 | 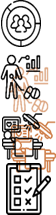 |
| --- | --- | --- | --- | --- | --- | --- | --- | --- | --- | --- |
| 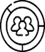 | 1 | 0.002 | 0.018 | 0.795 | 0.021 | 0.001 | 0.001 | 0.491 | 0.001 | 0.001 |
| 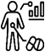 | 0.002 | 1 | 0.342 | 0.001 | 0.425 | 0.001 | 0.001 | 0.005 | 0.001 | 0.001 |
| 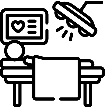 | 0.018 | 0.342 | 1 | 0.009 | 0.946 | 0.004 | 0.001 | 0.108 | 0.001 | 0.001 |
| 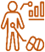 | 0.795 | 0.001 | 0.009 | 1 | 0.014 | 0.001 | 0.001 | 0.307 | 0.001 | 0.001 |
| 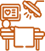 | 0.021 | 0.425 | 0.946 | 0.014 | 1 | 0.003 | 0.002 | 0.100 | 0.001 | 0.001 |
| 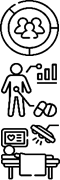 | 0.001 | 0.001 | 0.004 | 0.001 | 0.003 | 1 | 0.243 | 0.001 | 0.337 | 0.043 |
| 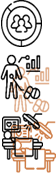 | 0.001 | 0.001 | 0.001 | 0.001 | 0.002 | 0.243 | 1 | 0.001 | 0.982 | 0.001 |
| 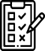 | 0.491 | 0.005 | 0.108 | 0.307 | 0.100 | 0.001 | 0.001 | 1 | 0.001 | 0.001 |
| 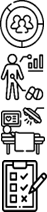 | 0.001 | 0.001 | 0.001 | 0.001 | 0.001 | 0.337 | 0.982 | 0.001 | 1 | 0.176 |
| 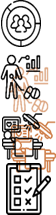 | 0.001 | 0.001 | 0.001 | 0.001 | 0.001 | 0.043 | 0.262 | 0.001 | 0.176 | 1 |

List of icons:

| Preoperative: Sociodemographic | 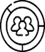 |
| --- | --- |
| Preoperative: Clinical | **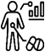** |
| Preoperative: Surgical | 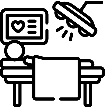 |
| Intraoperative : Clinical | **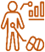** |
| Intraoperative : Surgical | **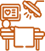** |
| Preoperative: Sociodemographic, Clinical, Surgical | 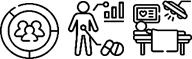 |
| Preoperative: Sociodemographic, Clinical, Surgical and Intraoperative: Clinical, Surgical | 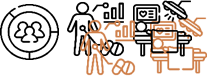 |
| Preoperative: Neuropsychological | **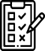** |
| Preoperative: Sociodemographic, Clinical, Surgical, Neuropsychological | 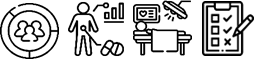 |
| Preoperative: Sociodemographic, Clinical, Surgical, Neuropsychological and  Intraoperative: Clinical, Surgical | 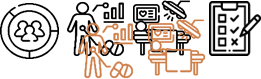 |

**Table S7.** Feature attributions of Preoperative: Sociodemographic, Clinical, Surgical, ranked by average absolute SHAP values (descending order).

| Feature | Average absolute SHAP value |
| --- | --- |
| 1. Preoperative: Types of surgery | 0.0658 |
| 1. Preoperative: Preoperative: Cardio-pulmonary bypass with heart-lung machine use | 0.0324 |
| 1. Preoperative: Location | 0.0304 |
| 1. Preoperative: ASA Physical Status Classification System at admission | 0.0224 |
| 1. Preoperative: Polypharmacy (number of medicines taken) at admission | 0.0195 |
| 1. Preoperative: Renal failure (creatinine clearance) at admission | 0.0177 |
| 1. Preoperative: Clinical Frailty Scale at admission | 0.0172 |
| 1. Preoperative: Age in years | 0.0159 |
| 1. Preoperative: Sex | 0.0140 |
| 1. Preoperative: Dose of preoperative Benzodiazepines use | 0.0130 |
| 1. Preoperative: Mini Nutritional Assessment (MNA) sum score at admission | 0.0115 |
| 1. Preoperative: Barthel Index sum score at admission | 0.0115 |
| 1. Preoperative: Sensory impairment at admission | 0.0105 |
| 1. Preoperative: Years of education in total | 0.0087 |
| 1. Preoperative: Multimorbidity (number of diseases reported) at admission | 0.0087 |
| 1. Preoperative: Auditory impairment at admission | 0.0084 |
| 1. Preoperative: Charlson Comorbidity Index at admission | 0.0078 |
| 1. Preoperative: Inhalation anesthesia | 0.0063 |
| 1. Preoperative: Hemoglobin level at admission | 0.0060 |
| 1. Preoperative: C-reactive protein at admission | 0.0059 |
| 1. Preoperative: Types of anesthesia | 0.0058 |
| 1. Preoperative: Alone living | 0.0049 |
| 1. Preoperative: Visual impairment at admission | 0.0045 |
| 1. Preoperative: Regional anesthesia | 0.0045 |
| 1. Preoperative: Sodium level at admission | 0.0036 |
| 1. Preoperative: Intravenous anesthesia | 0.0035 |
| 1. Preoperative: Previous delirium history | 0.0033 |
| 1. Preoperative: Cigarette consumption per day | 0.0028 |
| 1. Preoperative: Dose of preoperative Neuroleptics use | 0.0021 |
| 1. Preoperative: Dose of preoperative Propofol use | 0.0016 |
| 1. Preoperative: Dose of preoperative Opiates use | 0.0010 |
| 1. Preoperative: Alcohol consumption per month | 0 |
| 1. Preoperative: Preexisting dementia | 0 |

**Table S8.** Feature attributions of Preoperative: Sociodemographic, Clinical, Surgical and Intraoperative: Clinical, Surgical, ranked by average absolute SHAP values (descending order).

| Feature | Average absolute SHAP value |
| --- | --- |
| 1. Preoperative: Types of surgery | 0.0407 |
| 1. Intraoperative: Minutes of cut-to-suture time | 0.0307 |
| 1. Intraoperative: Minutes of anesthetic duration | 0.0209 |
| 1. Preoperative: Preoperative: Cardio-pulmonary bypass with heart-lung machine use | 0.0181 |
| 1. Preoperative: ASA Physical Status Classification System at admission | 0.0176 |
| 1. Intraoperative: Blood transfusion during surgery | 0.0162 |
| 1. Preoperative: Location | 0.0160 |
| 1. Intraoperative: Number of erythrocytes during surgery | 0.0138 |
| 1. Preoperative: Renal failure (creatinine clearance) at admission | 0.0137 |
| 1. Preoperative: Clinical Frailty Scale at admission | 0.0135 |
| 1. Preoperative: Age in years | 0.0132 |
| 1. Preoperative: Polypharmacy (number of medicines taken) at admission | 0.0132 |
| 1. Intraoperative: Dose of intraoperative Opiates use | 0.0122 |
| 1. Preoperative: Barthel Index sum score at admission | 0.0111 |
| 1. Preoperative: Sensory impairment at admission | 0.0100 |
| 1. Preoperative: Dose of preoperative Benzodiazepines use | 0.0092 |
| 1. Preoperative: Mini Nutritional Assessment (MNA) sum score at admission | 0.0083 |
| 1. Preoperative: Auditory impairment at admission | 0.0082 |
| 1. Intraoperative: Dose of intraoperative Propofol use | 0.0074 |
| 1. Preoperative: Sex | 0.0072 |
| 1. Intraoperative: Increase in creatine >0.3mg/dl during surgery | 0.007 |
| 1. Intraoperative: Dose of intraoperative Benzodiazepines use | 0.0069 |
| 1. Preoperative: Multimorbidity (number of diseases reported) at admission | 0.0065 |
| 1. Intraoperative: Drop in hemoglobin value >3g/dl during surgery | 0.0061 |
| 1. Preoperative: Years of education in total | 0.0058 |
| 1. Preoperative: Charlson Comorbidity Index at admission | 0.0058 |
| 1. Preoperative: Visual impairment at admission | 0.0048 |
| 1. Preoperative: C-reactive protein at admission | 0.0044 |
| 1. Preoperative: Hemoglobin level at admission | 0.0040 |
| 1. Preoperative: Types of anesthesia | 0.0036 |
| 1. Preoperative: Alone living | 0.0033 |
| 1. Preoperative: Previous delirium history | 0.0031 |
| 1. Preoperative: Inhalation anesthesia | 0.0027 |
| 1. Intraoperative: Blood pressure drop during surgery | 0.0023 |
| 1. Preoperative: Regional anesthesia | 0.0021 |
| 1. Preoperative: Sodium level at admission | 0.0020 |
| 1. Preoperative: Dose of preoperative Neuroleptics use | 0.0019 |
| 1. Preoperative: Cigarette consumption per day | 0.0018 |
| 1. Preoperative: Intravenous anesthesia | 0.0013 |
| 1. Preoperative: Dose of preoperative Propofol use | 0.0013 |
| 1. Preoperative: Dose of preoperative Opiates use | 0.0008 |
| 1. Intraoperative: Dose of intraoperative Neuroleptics use | 0.0007 |
| 1. Preoperative: Alcohol consumption per month | 0 |
| 1. Preoperative: Preexisting dementia | 0 |

**Table S9.** Feature attributions of Preoperative: Sociodemographic, Clinical, Surgical, Neuropsychological, ranked by average absolute SHAP values (descending order).

| Feature | Average absolute SHAP value |
| --- | --- |
| 1. Preoperative: Types of surgery | 0.0598 |
| 1. Preoperative: Preoperative: Cardio-pulmonary bypass with heart-lung machine use | 0.0337 |
| 1. Preoperative: MoCA Memory at admission | 0.0225 |
| 1. Preoperative: Trial Making Test part B (TMT-B) in seconds at admission | 0.0223 |
| 1. Preoperative: Location | 0.0216 |
| 1. Preoperative: ASA Physical Status Classification System at admission | 0.0181 |
| 1. Preoperative: Renal failure (creatinine clearance) at admission | 0.0142 |
| 1. Preoperative: Trial Making Test part A (TMT-A) in seconds at admission | 0.0132 |
| 1. Preoperative: Montreal Cognitive Assessment (MoCA) sum score at admission | 0.0130 |
| 1. Preoperative: Dose of preoperative Benzodiazepines use | 0.0125 |
| 1. Preoperative: Polypharmacy (number of medicines taken) at admission | 0.0107 |
| 1. Preoperative: MoCA Orientation at admission | 0.0100 |
| 1. Preoperative: Sex | 0.0096 |
| 1. Preoperative: Age in years | 0.0091 |
| 1. Preoperative: Clinical Frailty Scale at admission | 0.0077 |
| 1. Preoperative: Mini Nutritional Assessment (MNA) sum score at admission | 0.0073 |
| 1. Preoperative: Subjective memory impairment (SMI) at admission | 0.0070 |
| 1. Preoperative: Multimorbidity (number of diseases reported) at admission | 0.0064 |
| 1. Preoperative: Charlson Comorbidity Index at admission | 0.0062 |
| 1. Preoperative: Digit span (length) at admission | 0.0061 |
| 1. Preoperative: Inhalation anesthesia | 0.0060 |
| 1. Preoperative: Barthel Index sum score at admission | 0.0055 |
| 1. Preoperative: Years of education in total | 0.0054 |
| 1. Preoperative: Sensory impairment at admission | 0.0046 |
| 1. Preoperative: Regional anesthesia | 0.0045 |
| 1. Preoperative: MoCA Visuospatial/Executive at admission | 0.0042 |
| 1. Preoperative: MoCA Attention at admission | 0.0041 |
| 1. Preoperative: MoCA Abstraction at admission | 0.0041 |
| 1. Preoperative: Patient Health Questionnaire-4 (PHQ-4) at admission | 0.0039 |
| 1. Preoperative: Hemoglobin level at admission | 0.0039 |
| 1. Preoperative: Auditory impairment at admission | 0.0037 |
| 1. Preoperative: Types of anesthesia | 0.0036 |
| 1. Preoperative: PHQ-4 anxiety rating at admission | 0.0036 |
| 1. Preoperative: C-reactive protein at admission | 0.0032 |
| 1. Preoperative: PHQ-4 depression rating at admission | 0.0032 |
| 1. Preoperative: MoCA Language at admission | 0.0031 |
| 1. Preoperative: Alone living | 0.0026 |
| 1. Preoperative: Visual impairment at admission | 0.0024 |
| 1. Preoperative: Sodium level at admission | 0.0021 |
| 1. Preoperative: Cigarette consumption per day | 0.0020 |
| 1. Preoperative: Intravenous anesthesia | 0.0016 |
| 1. Preoperative: Previous delirium history | 0.0016 |
| 1. Preoperative: MoCA Naming at admission | 0.0014 |
| 1. Preoperative: Dose of preoperative Propofol use | 0.0012 |
| 1. Preoperative: Dose of preoperative Neuroleptics use | 0.0010 |
| 1. Preoperative: Dose of preoperative Opiates use | 0.0006 |
| 1. Preoperative: Alcohol consumption per month | 0 |
| 1. Preoperative: Preexisting dementia | 0 |

**Table S10.** Feature attributions of Preoperative: Sociodemographic, Clinical, Surgical, Neuropsychological and

Intraoperative: Clinical, Surgical, ranked by average absolute SHAP values (descending order).

| Feature | Average absolute SHAP value |
| --- | --- |
| 1. Intraoperative: Minutes of cut-to-suture time | 0.0403 |
| 1. Preoperative: Types of surgery | 0.0271 |
| 1. Intraoperative: Minutes of anesthetic duration | 0.0229 |
| 1. Preoperative: Trial Making Test part B (TMT-B) in seconds at admission | 0.0197 |
| 1. Preoperative: MoCA Memory at admission | 0.0194 |
| 1. Preoperative: Preoperative: Cardio-pulmonary bypass with heart-lung machine use | 0.0154 |
| 1. Preoperative: Location | 0.0137 |
| 1. Intraoperative: Number of erythrocytes during surgery | 0.0130 |
| 1. Intraoperative: Blood transfusion during surgery | 0.0126 |
| 1. Preoperative: Montreal Cognitive Assessment (MoCA) sum score at admission | 0.0123 |
| 1. Preoperative: ASA Physical Status Classification System at admission | 0.0120 |
| 1. Preoperative: Trial Making Test part A (TMT-A) in seconds at admission | 0.0108 |
| 1. Intraoperative: Dose of intraoperative Opiates use | 0.0098 |
| 1. Preoperative: Renal failure (creatinine clearance) at admission | 0.0096 |
| 1. Preoperative: MoCA Orientation at admission | 0.0088 |
| 1. Preoperative: Dose of preoperative Benzodiazepines use | 0.0085 |
| 1. Preoperative: Polypharmacy (number of medicines taken) at admission | 0.0079 |
| 1. Intraoperative: Dose of intraoperative Propofol use | 0.0077 |
| 1. Preoperative: Age in years | 0.0076 |
| 1. Preoperative: Sex | 0.0065 |
| 1. Preoperative: Clinical Frailty Scale at admission | 0.0063 |
| 1. Preoperative: Subjective memory impairment (SMI) at admission | 0.0061 |
| 1. Intraoperative: Dose of intraoperative Benzodiazepines use | 0.0059 |
| 1. Intraoperative: Drop in hemoglobin value >3g/dl during surgery | 0.0055 |
| 1. Preoperative: Mini Nutritional Assessment (MNA) sum score at admission | 0.0054 |
| 1. Preoperative: Multimorbidity (number of diseases reported) at admission | 0.0052 |
| 1. Preoperative: Barthel Index sum score at admission | 0.0050 |
| 1. Preoperative: Digit span (length) at admission | 0.0049 |
| 1. Intraoperative: Increase in creatine >0.3mg/dl during surgery | 0.0048 |
| 1. Preoperative: Charlson Comorbidity Index at admission | 0.0046 |
| 1. Preoperative: Years of education in total | 0.0044 |
| 1. Preoperative: Sensory impairment at admission | 0.0043 |
| 1. Preoperative: MoCA Visuospatial/Executive at admission | 0.0043 |
| 1. Preoperative: MoCA Attention at admission | 0.0035 |
| 1. Preoperative: Patient Health Questionnaire-4 (PHQ-4) at admission | 0.0032 |
| 1. Preoperative: Hemoglobin level at admission | 0.0030 |
| 1. Preoperative: Auditory impairment at admission | 0.0029 |
| 1. Preoperative: MoCA Language at admission | 0.0029 |
| 1. Preoperative: C-reactive protein at admission | 0.0027 |
| 1. Preoperative: MoCA Abstraction at admission | 0.0026 |
| 1. Preoperative: PHQ-4 depression rating at admission | 0.0026 |
| 1. Preoperative: PHQ-4 anxiety rating at admission | 0.0025 |
| 1. Preoperative: Types of anesthesia | 0.0025 |
| 1. Preoperative: Visual impairment at admission | 0.0024 |
| 1. Preoperative: Inhalation anesthesia | 0.0021 |
| 1. Preoperative: Alone living | 0.0018 |
| 1. Preoperative: Regional anesthesia | 0.0016 |
| 1. Preoperative: Previous delirium history | 0.0016 |
| 1. Intraoperative: Blood pressure drop during surgery | 0.0016 |
| 1. Preoperative: MoCA Naming at admission | 0.0012 |
| 1. Preoperative: Cigarette consumption per day | 0.0012 |
| 1. Preoperative: Sodium level at admission | 0.0011 |
| 1. Preoperative: Dose of preoperative Neuroleptics use | 0.0011 |
| 1. Preoperative: Dose of preoperative Propofol use | 0.0010 |
| 1. Preoperative: Intravenous anesthesia | 0.0008 |
| 1. Preoperative: Dose of preoperative Opiates use | 0.0005 |
| 1. Intraoperative: Dose of intraoperative Neuroleptics use | 0.0003 |
| 1. Preoperative: Alcohol consumption per month | 0 |
| 1. Preoperative: Preexisting dementia | 0 |

**Table S11.** Model performances with four classifiers

| Model: mean area under the receiver operating characteristic curve | Linear SVC | Random Forest | Logistic Regression | Gradient boosting |
| --- | --- | --- | --- | --- |
| Preoperative: Sociodemographic | 0.509 | 0.587 | **0.647** | 0.583 |
| Preoperative: Clinical | 0.475 | 0.693 | **0.705** | 0.652 |
| Preoperative: Surgical | 0.492 | **0.665** | 0.652 | **0.665** |
| Intraoperative : Clinical | 0.503 | 0.577 | **0.628** | 0.586 |
| Intraoperative : Surgical | 0.544 | 0.664 | **0.715** | 0.653 |
| Preoperative: Sociodemographic, Clinical, Surgical | 0.722 | **0.760** | 0.753 | 0.745 |
| Preoperative: Sociodemographic, Clinical, Surgical and  Intraoperative: Clinical, Surgical | 0.769 | **0.785** | 0.769 | 0.755 |
| Preoperative: Neuropsychological | 0.575 | 0.616 | **0.646** | 0.570 |
| Preoperative: Sociodemographic, Clinical, Surgical, Neuropsychological | 0.783 | **0.791** | 0.785 | 0.753 |
| Preoperative: Sociodemographic, Clinical, Surgical, Neuropsychological and Intraoperative: Clinical, Surgical | 0.796 | **0.809** | 0.798 | 0.781 |

**Bold** = best-performing classifier of each model.

**Table S12.** Sensitivity analyses of hyperparameters tunning for Preoperative: Sociodemographic, Clinical, Surgical

| Model (hyperparameter) : mean | ROC | PRC | Sen | Spe | Prec | Rec | BA |
| --- | --- | --- | --- | --- | --- | --- | --- |
| Linear SVC (C=0.1) | 0.719 | 0.428 | 10.67 | 97.68 | 57.97 | 10.67 | 54.17 |
| Linear SVC (C=1) | 0.723 | 0.439 | 15.20 | 96.48 | 56.44 | 15.20 | 55.84 |
| Linear SVC (C=10) | 0.722 | 0.437 | 15.47 | 96.32 | 55.77 | 15.47 | 55.89 |
| Linear SVC (balanced) | 0.752 | 0.499 | 66.40 | 71.66 | 41.29 | 66.40 | 69.03 |
| Random Forest (n=100) | 0.756 | 0.495 | 24.27 | 94.64 | 57.59 | 24.27 | 59.45 |
| Random Forest (n=200) | 0.760 | 0.513 | 28.53 | 94.80 | 62.21 | 28.53 | 61.66 |
| Random Forest (n=300) | 0.764 | 0.509 | 26.67 | 94.40 | 58.82 | 26.67 | 60.53 |
| Random Forest (depth=10) | **0.767** | 0.501 | 24.27 | 94.72 | 57.96 | 24.27 | 59.49 |
| Random Forest (depth=20) | 0.764 | 0.514 | 27.47 | 94.56 | 60.23 | 27.47 | 61.01 |
| Random Forest (balanced) | 0.762 | 0.484 | 23.47 | 94.96 | 58.28 | 23.47 | 59.21 |
| Logistic Regression (C=0.1) | 0.756 | 0.504 | 27.73 | 94.16 | 58.76 | 27.73 | 60.94 |
| Logistic Regression (C=1) | 0.753 | 0.497 | 29.60 | 93.43 | 57.51 | 29.6 | 61.52 |
| Logistic Regression (C=10) | 0.752 | 0.496 | 30.13 | 93.19 | 57.07 | 30.13 | 61.66 |
| Logistic Regression (L1) | 0.756 | 0.504 | 29.33 | 93.67 | 58.20 | 29.33 | 61.50 |
| Logistic Regression (balanced) | 0.749 | 0.486 | **68.53** | 70.14 | 40.79 | **68.53** | **69.33** |
| Gradient boosting (default) | 0.745 | 0.515 | 37.33 | 91.43 | 56.68 | 37.33 | 64.38 |
| Gradient boosting (lr=0.01) | 0.758 | 0.511 | 14.67 | **98.24** | **71.43** | 14.67 | 56.45 |
| Gradient boosting (lr=0.1) | 0.758 | 0.533 | 33.87 | 92.79 | 58.53 | 33.87 | 63.33 |
| Gradient boosting (n=200) | 0.749 | 0.514 | 36.53 | 90.87 | 54.58 | 36.53 | 63.7 |
| Gradient boosting (depth=3) | 0.750 | **0.535** | 35.47 | 92.79 | 59.64 | 35.47 | 64.13 |
| Gradient boosting (depth=9) | 0.736 | 0.487 | 34.13 | 90.71 | 52.46 | 34.13 | 62.42 |

ROC = area under the receiver operating characteristic curve; PRC= area under the precision-recall curve; Sen= sensitivity; Spe=specificity; Prec= precision; Rec= recall; BA= balanced accuracy, **Bold** = best-performing model for each performance metric.

**Table S13.** Sensitivity analyses of hyperparameters tunning for Preoperative: Sociodemographic, Clinical, Surgical and Intraoperative: Clinical, Surgical

| Model with hyperparameters | ROC | PRC | Sen | Spe | Prec | Rec | BA |
| --- | --- | --- | --- | --- | --- | --- | --- |
| Linear SVC (C=0.1) | 0.771 | 0.506 | 27.20 | 94.48 | 59.65 | 27.20 | 60.84 |
| Linear SVC (C=1) | 0.770 | 0.508 | 28.53 | 94.32 | 60.11 | 28.53 | 61.42 |
| Linear SVC (C=10) | 0.767 | 0.504 | 28.53 | 94.40 | 60.45 | 28.53 | 61.46 |
| Linear SVC (balanced) | 0.771 | 0.516 | **69.60** | 72.14 | 42.86 | **69.60** | **70.87** |
| Random Forest (n=100) | 0.790 | **0.533** | 27.20 | 95.92 | **66.67** | 27.20 | 61.56 |
| Random Forest (n=200) | **0.791** | 0.531 | 27.20 | 95.20 | 62.96 | 27.20 | 61.20 |
| Random Forest (n=300) | 0.789 | 0.528 | 26.93 | 95.44 | 63.92 | 26.93 | 61.18 |
| Random Forest (depth=10) | 0.780 | 0.519 | 24.53 | 95.12 | 60.13 | 24.53 | 59.82 |
| Random Forest (depth=20) | 0.785 | 0.524 | 26.67 | 94.72 | 60.24 | 26.67 | 60.69 |
| Random Forest (balanced) | 0.789 | 0.517 | 24.00 | 94.88 | 58.44 | 24.00 | 59.44 |
| Logistic Regression (C=0.1) | 0.774 | 0.513 | 31.73 | 92.87 | 57.21 | 31.73 | 62.30 |
| Logistic Regression (C=1) | 0.769 | 0.503 | 33.33 | 92.15 | 56.05 | 33.33 | 62.74 |
| Logistic Regression (C=10) | 0.769 | 0.502 | 33.60 | 92.23 | 56.50 | 33.60 | 62.92 |
| Logistic Regression (L1) | 0.773 | 0.510 | 32.80 | 92.31 | 56.16 | 32.80 | 62.56 |
| Logistic Regression (balanced) | 0.768 | 0.502 | 68.00 | 73.18 | 43.22 | 68.00 | 70.59 |
| Gradient boosting (default) | 0.755 | 0.505 | 34.40 | 90.95 | 53.31 | 34.40 | 62.68 |
| Gradient boosting (lr=0.01) | 0.775 | 0.514 | 13.87 | **97.84** | 65.82 | 13.87 | 55.85 |
| Gradient boosting (lr=0.1) | 0.772 | **0.533** | 36.53 | 92.07 | 58.05 | 36.53 | 64.30 |
| Gradient boosting (n=200) | 0.763 | 0.528 | 37.33 | 91.99 | 58.33 | 37.33 | 64.66 |
| Gradient boosting (depth=3) | 0.769 | **0.533** | 36.27 | 91.51 | 56.20 | 36.27 | 63.89 |
| Gradient boosting (depth=9) | 0.763 | 0.503 | 34.67 | 91.59 | 55.32 | 34.67 | 63.13 |

ROC = area under the receiver operating characteristic curve; PRC= area under the precision-recall curve; Sen= sensitivity; Spe=specificity; Prec= precision; Rec= recall; BA= balanced accuracy, **Bold** = best-performing model for each performance metric.

**Table S14.** Sensitivity analyses of hyperparameters tunning for Preoperative: Sociodemographic, Clinical, Surgical, Neuropsychological

| Model with hyperparameters | ROC | PRC | Sen | Spe | Prec | Rec | BA |
| --- | --- | --- | --- | --- | --- | --- | --- |
| Linear SVC (C=0.1) | 0.785 | 0.539 | 33.33 | 93.59 | 60.98 | 33.33 | 63.46 |
| Linear SVC (C=1) | 0.783 | 0.541 | 32.27 | 93.27 | 59.02 | 32.27 | 62.77 |
| Linear SVC (C=10) | 0.782 | 0.539 | 32.53 | 93.11 | 58.65 | 32.53 | 62.82 |
| Linear SVC (balanced) | 0.781 | **0.557** | **69.33** | 72.70 | 43.26 | **69.33** | **71.02** |
| Random Forest (n=100) | **0.790** | 0.546 | 30.13 | 95.20 | **65.32** | 30.13 | 62.66 |
| Random Forest (n=200) | **0.790** | 0.552 | 31.20 | 94.48 | 62.90 | 31.20 | 62.84 |
| Random Forest (n=300) | **0.790** | 0.546 | 29.07 | 94.80 | 62.64 | 29.07 | 61.93 |
| Random Forest (depth=10) | 0.788 | 0.539 | 28.80 | 94.88 | 62.79 | 28.80 | 61.84 |
| Random Forest (depth=20) | 0.782 | 0.534 | 32.53 | 94.16 | 62.56 | 32.53 | 63.34 |
| Random Forest (balanced) | 0.788 | 0.529 | 24.80 | 95.92 | 64.58 | 24.80 | 60.36 |
| Logistic Regression (C=0.1) | 0.789 | **0.557** | 36.53 | 93.11 | 61.43 | 36.53 | 64.82 |
| Logistic Regression (C=1) | 0.785 | 0.549 | 37.60 | 92.39 | 59.75 | 37.60 | 65.00 |
| Logistic Regression (C=10) | 0.783 | 0.542 | 38.13 | 92.31 | 59.83 | 38.13 | 65.22 |
| Logistic Regression (L1) | 0.788 | **0.557** | 37.07 | 92.55 | 59.91 | 37.07 | 64.81 |
| Logistic Regression (balanced) | 0.781 | 0.546 | 68.00 | 72.46 | 42.57 | 68.00 | 70.23 |
| Gradient boosting (default) | 0.753 | 0.495 | 38.93 | 91.67 | 58.40 | 38.93 | 65.30 |
| Gradient boosting (lr=0.01) | 0.778 | 0.504 | 12.53 | **97.28** | 58.02 | 12.53 | 54.91 |
| Gradient boosting (lr=0.1) | 0.769 | 0.510 | 37.33 | 92.39 | 59.57 | 37.33 | 64.86 |
| Gradient boosting (n=200) | 0.769 | 0.516 | 38.67 | 91.59 | 58.00 | 38.67 | 65.13 |
| Gradient boosting (depth=3) | 0.758 | 0.499 | 39.47 | 91.75 | 58.96 | 39.47 | 65.61 |
| Gradient boosting (depth=9) | 0.767 | 0.517 | 37.87 | 92.15 | 59.17 | 37.87 | 65.01 |

ROC = area under the receiver operating characteristic curve; PRC= area under the precision-recall curve; Sen= sensitivity; Spe=specificity; Prec= precision; Rec= recall; BA= balanced accuracy, **Bold** = best-performing model for each performance metric.

**Table S15.** Sensitivity analyses of hyperparameters tunning for Preoperative: Sociodemographic, Clinical, Surgical, Neuropsychological and Intraoperative: Clinical, Surgical

| Model with hyperparameters | ROC | PRC | Sen | Spe | Prec | Rec | BA |
| --- | --- | --- | --- | --- | --- | --- | --- |
| Linear SVC (C=0.1) | 0.801 | 0.556 | 36.27 | 92.95 | 60.71 | 36.27 | 64.61 |
| Linear SVC (C=1) | 0.797 | 0.550 | 36.53 | 92.47 | 59.31 | 36.53 | 64.50 |
| Linear SVC (C=10) | 0.796 | 0.550 | 37.07 | 92.31 | 59.15 | 37.07 | 64.69 |
| Linear SVC (balanced) | 0.793 | 0.568 | 68.53 | 74.54 | 44.70 | 68.53 | 71.54 |
| Random Forest (n=100) | 0.802 | 0.556 | 29.87 | 94.32 | 61.20 | 29.87 | 62.09 |
| Random Forest (n=200) | **0.809** | 0.567 | 32.80 | 95.04 | **66.49** | 32.80 | 63.92 |
| Random Forest (n=300) | 0.808 | 0.563 | 31.47 | 94.80 | 64.48 | 31.47 | 63.13 |
| Random Forest (depth=10) | 0.803 | 0.547 | 32.00 | 94.72 | 64.52 | 32.00 | 63.36 |
| Random Forest (depth=20) | 0.808 | 0.560 | 29.33 | 95.44 | 65.87 | 29.33 | 62.38 |
| Random Forest (balanced) | 0.806 | 0.540 | 25.33 | 95.60 | 63.33 | 25.33 | 60.46 |
| Logistic Regression (C=0.1) | 0.804 | **0.572** | 37.87 | 92.39 | 59.92 | 37.87 | 65.13 |
| Logistic Regression (C=1) | 0.798 | 0.565 | 38.40 | 92.23 | 59.75 | 38.40 | 65.32 |
| Logistic Regression (C=10) | 0.796 | 0.558 | 38.93 | 92.15 | 59.84 | 38.93 | 65.54 |
| Logistic Regression (L1) | 0.802 | 0.570 | 38.40 | 92.31 | 60.00 | 38.40 | 65.36 |
| Logistic Regression (balanced) | 0.795 | 0.562 | **69.07** | 75.50 | 45.84 | **69.07** | **72.28** |
| Gradient boosting (default) | 0.781 | 0.543 | 37.87 | 91.59 | 57.49 | 37.87 | 64.73 |
| Gradient boosting (lr=0.01) | 0.786 | 0.511 | 14.67 | **97.44** | 63.22 | 14.67 | 56.05 |
| Gradient boosting (lr=0.1) | 0.797 | 0.552 | 37.87 | 92.31 | 59.66 | 37.87 | 65.09 |
| Gradient boosting (n=200) | 0.792 | 0.551 | 37.07 | 92.07 | 58.40 | 37.07 | 64.57 |
| Gradient boosting (depth=3) | 0.784 | 0.531 | 38.40 | 91.11 | 56.47 | 38.40 | 64.76 |
| Gradient boosting (depth=9) | 0.789 | 0.547 | 39.47 | 92.07 | 59.92 | 39.47 | 65.77 |

ROC = area under the receiver operating characteristic curve; PRC= area under the precision-recall curve; Sen= sensitivity; Spe=specificity; Prec= precision; Rec= recall; BA= balanced accuracy, **Bold** = best-performing model for each performance metric.

**Table S16.** Sensitivity analyses of performance difference in the area under the receiver operating characteristic curve between models with additional intervention allocation information: 1000 permutation tests.

| *P* | 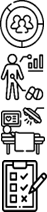 | 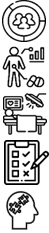 | 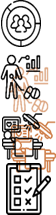 | 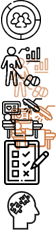 |
| --- | --- | --- | --- | --- |
| 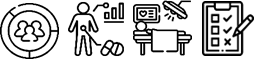 | 1 | 0.863 | 0.176 | 0.412 |
| 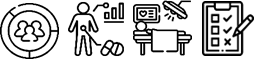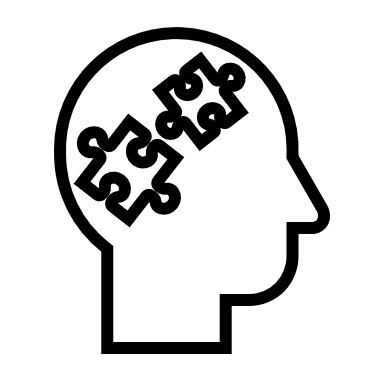 | 0.863 | 1 | 0.147 | 0.313 |
| 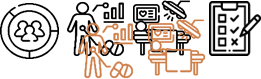 | 0.176 | 0.147 | 1 | 0.624 |
| 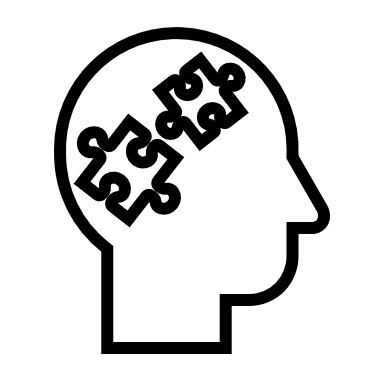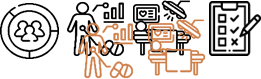 | 0.412 | 0.313 | 0.624 | 1 |

List of icons:

| Preoperative: Sociodemographic, Clinical, Surgical, Neuropsychological | 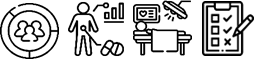 |
| --- | --- |
| Preoperative: Sociodemographic, Clinical, Surgical, Neuropsychological, Intervention allocation | 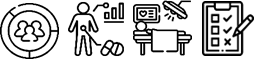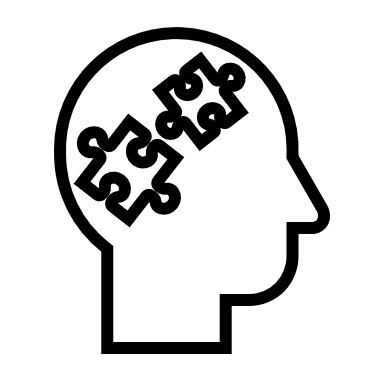 |
| Preoperative: Sociodemographic, Clinical, Surgical, Neuropsychological and  Intraoperative: Clinical, Surgical | 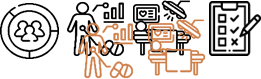 |
| Preoperative: Sociodemographic, Clinical, Surgical, Neuropsychological, Intervention allocation and Intraoperative: Clinical, Surgical | 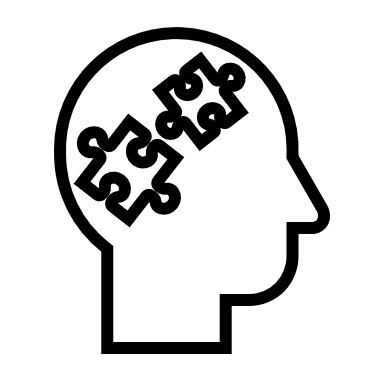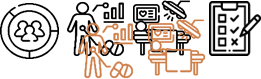 |

**Table S17.** Sensitivity analyses of labels oversampling with the Synthetic Minority Oversampling Technique (SMOTE)

| \| Model: mean \| Oversampling \| ROC \| BA \| \| --- \| --- \| --- \| --- \| \| Preoperative: Sociodemographic \| None \| 0.589 \| 52.87 \| \| SMOTE \| 0.591 \| 54.26 \| \| Preoperative: Clinical \| None \| 0.692 \| 57.03 \| \| SMOTE \| 0.676 \| 58.12 \| \| Preoperative: Surgical \| None \| 0.664 \| 50.00 \| \| SMOTE \| 0.658 \| 65.91 \| \| Intraoperative: Clinical \| None \| 0.576 \| 55.22 \| \| SMOTE \| 0.558 \| 54.66 \| \| Intraoperative: Surgical \| None \| 0.668 \| 59.73 \| \| SMOTE \| 0.664 \| 60.58 \| \| Preoperative: Sociodemographic, Clinical, Surgical \| None \| 0.765 \| 60.82 \| \| SMOTE \| 0.761 \| 62.85 \| \| Preoperative: Sociodemographic, Clinical, Surgical and  Intraoperative: Clinical, Surgical \| None \| 0.791 \| 60.92 \| \| SMOTE \| 0.794 \| 64.80 \| \| Preoperative: Neuropsychological \| None \| 0.626 \| 54.49 \| \| SMOTE \| 0.612 \| 55.56 \| \| Preoperative: Sociodemographic, Clinical, Surgical, Neuropsychological \| None \| 0.784 \| 61.41 \| \| SMOTE \| 0.782 \| 66.40 \| \| Preoperative: Sociodemographic, Clinical, Surgical, Neuropsychological and Intraoperative: Clinical, Surgical \| None \| 0.805 \| 62.85 \| \| SMOTE \| 0.804 \| 66.66 \|   ROC = area under the receiver operating characteristic curve; BA= balanced accuracy |
| --- | --- | --- | --- | --- | --- | --- | --- | --- | --- | --- | --- | --- | --- | --- | --- | --- | --- | --- | --- | --- | --- | --- | --- | --- | --- | --- | --- | --- | --- | --- | --- | --- | --- | --- | --- | --- | --- | --- | --- | --- | --- | --- | --- | --- | --- | --- | --- | --- | --- | --- | --- | --- | --- | --- | --- | --- | --- | --- | --- | --- | --- | --- | --- | --- | --- | --- | --- | --- | --- | --- | --- | --- | --- | --- |
